# Exploring the dynamics of monkeypox transmission with data-driven methods and a deterministic model

**DOI:** 10.3389/fepid.2024.1334964

**Published:** 2024-05-22

**Authors:** Haridas K. Das

**Affiliations:** ^1^ Department of Mathematics, Oklahoma State University, Stillwater, OK, United States; ^2^ Department of Mathematics, Dhaka University, Dhaka, Bangladesh

**Keywords:** epidemiology, Mpox, univariate time series, deterministic model, neural networks

## Abstract

**Introduction:**

Mpox (formerly monkeypox) is an infectious disease that spreads mostly through direct contact with infected animals or people's blood, bodily fluids, or cutaneous or mucosal lesions. In light of the global outbreak that occurred in 2022–2023, in this paper, we analyzed global Mpox univariate time series data and provided a comprehensive analysis of disease outbreaks across the world, including the USA with Brazil and three continents: North America, South America, and Europe. The novelty of this study is that it delved into the Mpox time series data by implementing the data-driven methods and a mathematical model concurrently—an aspect not typically addressed in the existing literature. The study is also important because implementing these models concurrently improved our predictions' reliability for infectious diseases.

**Methods:**

We proposed a traditional compartmental model and also implemented deep learning models (1D- convolutional neural network (CNN), long-short term memory (LSTM), bidirectional LSTM (BiLSTM), hybrid CNN-LSTM, and CNN-BiLSTM) as well as statistical time series models: autoregressive integrated moving average (ARIMA) and exponential smoothing on the Mpox data. We also employed the least squares method fitting to estimate the essential epidemiological parameters in the proposed deterministic model.

**Results:**

The primary finding of the deterministic model is that vaccination rates can flatten the curve of infected dynamics and influence the basic reproduction number. Through the numerical simulations, we determined that increased vaccination among the susceptible human population is crucial to control disease transmission. Moreover, in case of an outbreak, our model showed the potential for epidemic control by adjusting the key epidemiological parameters, namely the baseline contact rate and the proportion of contacts within the human population. Next, we analyzed data-driven models that contribute to a comprehensive understanding of disease dynamics in different locations. Additionally, we trained models to provide short-term (eight-week) predictions across various geographical locations, and all eight models produced reliable results.

**Conclusion:**

This study utilized a comprehensive framework to investigate univariate time series data to understand the dynamics of Mpox transmission. The prediction showed that Mpox is in its die-out situation as of July 29, 2023. Moreover, the deterministic model showed the importance of the Mpox vaccination in mitigating the Mpox transmission and highlighted the significance of effectively adjusting key epidemiological parameters during outbreaks, particularly the contact rate in high-risk groups.

## 1 Introduction

In the past 20 years, the world has experienced several epidemics that occurred throughout history, as mentioned in the previous studies by Piret and Boivin (1). Although many diseases, such as H1N1 influenza, COVID-19, and the current Mpox virus, have posed a major public health threat (2), mathematical and data-driven modeling can help to control and prevent the spread of disease by understanding the dynamics of disease. Moreover, a model can predict the potential course of disease progression and offer the best and most practical solutions to prevent pandemics when it incorporates fundamental features (3). In the initial stages of an outbreak, researchers paid widespread attention to mathematical modeling, with the general anticipation that deterministic models would accurately forecast the pandemic’s course, as it helps to understand the exponential increase of infections. This resurgence of disease modeling is not new. However, data-driven modeling opens a new era to explore the dynamics of diseases using real-world data. While numerous data-driven models have interpretability issues, focusing solely on a single data-driven approach may mislead decision-makers. In spite of having some limitations, data-driven techniques have increased their popularity in disease modeling for controlling epidemics using real-world data (4), particularly in epidemiological data. On the other hand, the deterministic model offers a structured and mathematically tractable framework for understanding the spread of infectious diseases, helping disease control, and enabling informed public health decisions. Therefore, integrating mathematical models and data-driven approaches becomes imperative for effective public health data implementation and control strategies, allowing for a more comprehensive and adaptive response to dynamic health challenges.

Mpox is a viral zoonotic infection disease caused by the monkeypox virus, and its transmission is considered one of the threats to human health, which may happen due to increased animal-to-human contact (5), human-to-human transmission (6), and climate change as it influences the environment of their vectors (7). More specifically, transmission through environmental factors can occur when a person or an animal touches surfaces or materials recently contaminated with the virus, whether from infected humans or animals (8). Another zoonotic virus related to Mpox has also been found to be transmitted from animals to humans (9). The virus is a member of the genus Orthopoxvirus (10, 11), and it is related to smallpox (12), but it is generally less severe in humans (13). The disease was first identified in 1958 during an outbreak in an animal facility in Copenhagen, Denmark (14). It was later recognized as a cause of human illness in 1970 in the Democratic Republic of Congo (14–16). The natural reservoir of the virus is believed to be rodents, such as squirrels and prairie dogs, but it has also been identified in other animals, including monkeys, porcupines, and Gambian giant rats (13). However, the ongoing Mpox virus is an emergent human pathogen recently studied by Emily and Sassine (17), Khan et al. (18), and John et al. (19). It has been found that the disease spreads from person to person through close contact with respiratory or other bodily fluids (13). The symptoms of Mpox include fever, headache, muscle aches, and a rash that spreads from the face to other parts of the body and eventually forms a scab before falling off (13). As of July 10, 2023, no specific treatment has been approved for Mpox, but the disease can be prevented through vaccination proposed by Andrea et al. (11), avoiding close contact with infected animals or people, and generally practicing good hygiene (13).

Recently, Kanj et al. (20), Hasan and Saeed (21), and Jeta et al. (22) reviewed nineteen diseases, of which nine employed compartments compartmental (deterministic) models to analyze the transmission dynamics of the Mpox virus in populations comprising both humans and non-humans. These studies explored variations of the classical SIR (Susceptible-Infected-Recovered) vector-borne models (23–25) within the context of human and non-human interaction and utilized them to study Mpox disease transmission. Incorporating vaccination classes (26) is essential for the disease’s accurate representation of progression and incubation. Moreover, the lessons from COVID-19 highlight that vaccinating susceptible populations can address the threats of Mpox and other emerging infectious diseases. Thus, we attempted to introduce a Mpox vaccination class and integrate the smallpox vaccine or dose-1 vaccine into the deterministic model proposed by Mesady et al. (27), Yuan et al. (24), and Esteban et al. (25) to provide human protection strategies along with the model fitting for reported Mpox cases across locations.

The rapid spread of Mpox has disrupted the globe when the COVID-19 disease transmission is declining. During this time, only a few articles have been published on detecting Mpox disease using deep learning on the image data (28). Many articles utilize machine learning techniques to forecast COVID-19 (29, 30) time series such as extreme machine learning (ELM), multilayer perceptron (MLP), long short-term memory (LSTM) networks, gated recurrent unit (GRU), convolution neural network (CNN), and deep neural network (DNN) methods on time series data used to forecast COVID-19 cases and predict a possible ending point of the outbreak. Although LSTM is the most popular neural network model for time series prediction, CNNs can also perform this task effectively. Moreover, CNNs have been shown to outperform RNNs in various tasks involving sequential data (31). CNNs can also learn long-term dependencies in time series data through a combination of multiple 1D convolution layers. In 2022, Ketu and Mishra (19) used CNN-LSTM, a hybrid deep-learning prediction model, to forecast the COVID-19 pandemic across India. While some analytical studies have used the Mpox disease transmission models for understanding the dynamics of this disease (2, 27), less attention has been given to predicting the Mpox virus infection using data-driven modeling (28, 32).

In this study, we forecast Mpox disease transmission for short-term predictions across diverse geographical areas by utilizing concurrently the deterministic modeling and advanced deep learning techniques (including 1D-CNN, LSTM, BiLSTM, hybrid CNN-LSTM, and CNN-BiLSTM) alongside statistical time series models like ARIMA and exponential smoothing. We employed a deterministic model and ran simulations to gain valuable insights into the dynamics of the Mpox outbreak and evaluate the impact of vaccination on disease control. Our analysis incorporated real Mpox data from ourworldindata (33), which helped to identify the disease spread across diverse global locations using our epidemiological model. Furthermore, we estimate critical epidemiological parameters through data fitting within the deterministic model. The consistent outcomes from the eight models utilized in this study indicate reliable results. Moreover, our study highlights the significance of time series models and demonstrates that vaccination rates have the potential to “flatten the curve” of infected cases and influence the basic reproduction number. Increased vaccination among susceptible populations is pivotal in effectively managing disease transmission. While the threat of outbreaks persists, reducing contact rates among high-risk groups can mitigate the disease’s impact in specific regions or communities. Importantly, our eight employed models provide an eight-week short-term prediction showing that Mpox is currently in a declining phase. These findings contribute to a comprehensive understanding of disease dynamics across various locations, guiding targeted intervention strategies for controlling and mitigating infectious disease outbreaks.

The remainder of this paper is divided into six sections. Section 1. provides an account of the datasets and summarizes the global and regional Mpox cases. Section 3. briefly describes deterministic and data-driven models. Section 4. implements the univariate Mpox dataset in the time series models and fits the deterministic model to find the epidemiological parameters. Fitting the data is performed using MATLAB and its optimization toolbox, such as *fmincon*. Section 5. concludes with a discussion, and the conclusion is drawn in Section 6.

## 2 Materials

**Figure 1:**
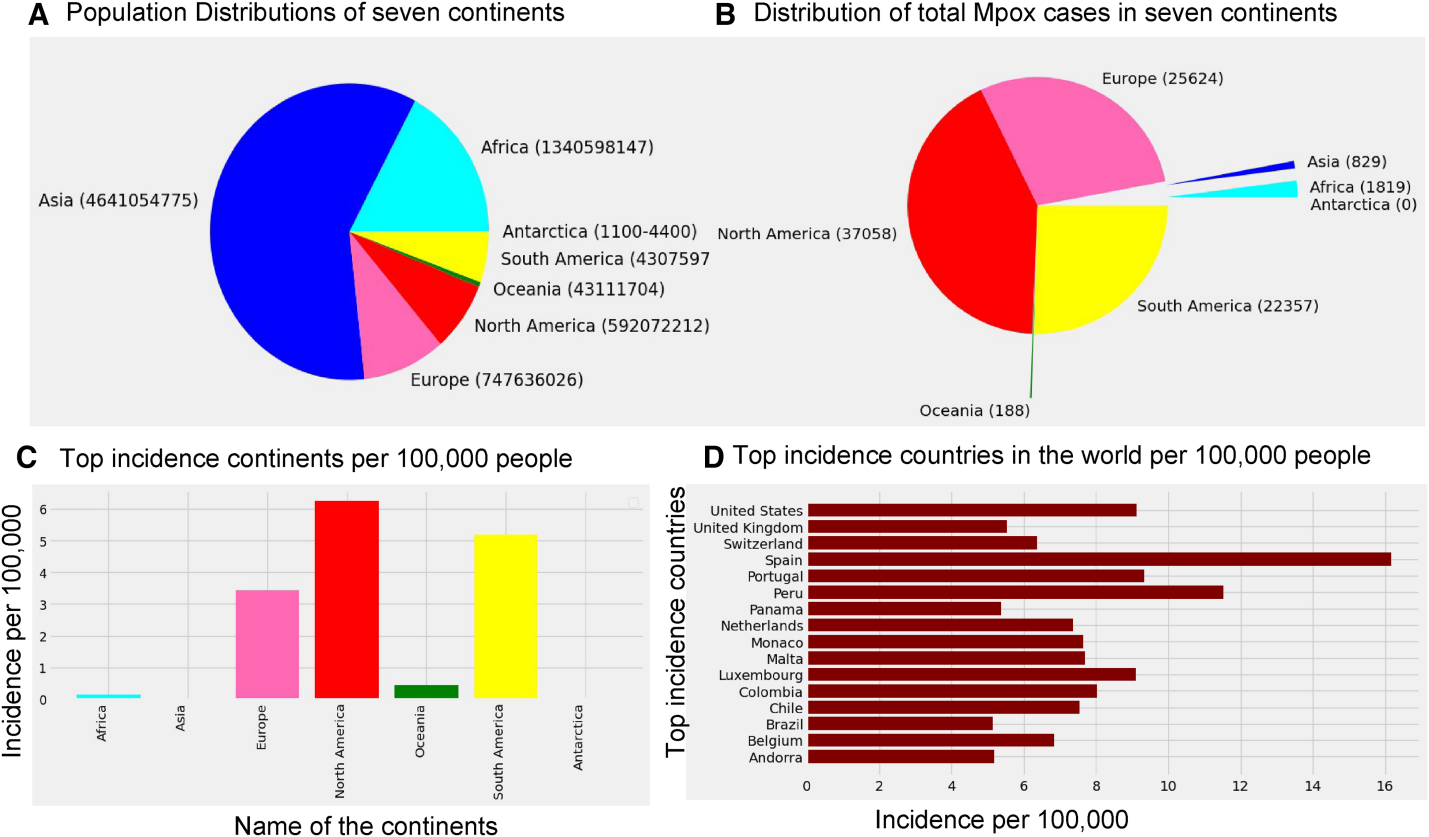
Distribution of the global population sizes, Mpox cases, and incidence: Panel (**A**) displays the population of each continent, where the world population is 7,795,237,030, and panel (**B**) shows the distribution of total Mpox cases in seven continents by the pie chart. Panel (**C**) presents incidence per 100 k individuals across the seven continents, where the maximum incidence occurs in North America (6.26 per 100 k), South America (5.19 per 100 k), and Europe (3.43 per 100 k). Panel (**D**) shows that Spain has a higher incidence of Mpox cases (greater than 15 per 100 k) compared to the other countries. Specifically, USA, Portugal, and Luxembourg maintain an incidence rate of 8 cases per 100 k, while the incidence rate in the remaining countries is below 8 per 100 k.

According to the Mpox data ourworldindata (33), the current Mpox virus spread globally, surpassing previous outbreaks and raising severe public health concerns in 2022–2023 by spreading to many regions (112 countries) worldwide. Excluding 27 cases reported before May 1, 2022, our study focuses on ourworldindata (33), which spans from May 1, 2022, to May 31, 2023, comprising 87,875 global new confirmed cases with 143 deaths. Moreover, this study employs various methodologies to predict the trajectory of the 2022–2023 Mpox epidemic in different regions, which is later verified with an additional eight weeks of Mpox cases from June 2023 to July 2023. The current transmission of Mpox has been reported on all continents except for Antarctica (Figure 1), as of May 31, 2023. The continents reporting the most cases are mainly North America (37,058), Europe (25,624), and the South Americas (22,357). Like coronavirus disease 2019 (COVID-19), the United States of America still leads the total confirmed cases at 30,225 as of May 31.

### 2.1 Mpox dataset description

**Figure 2:**
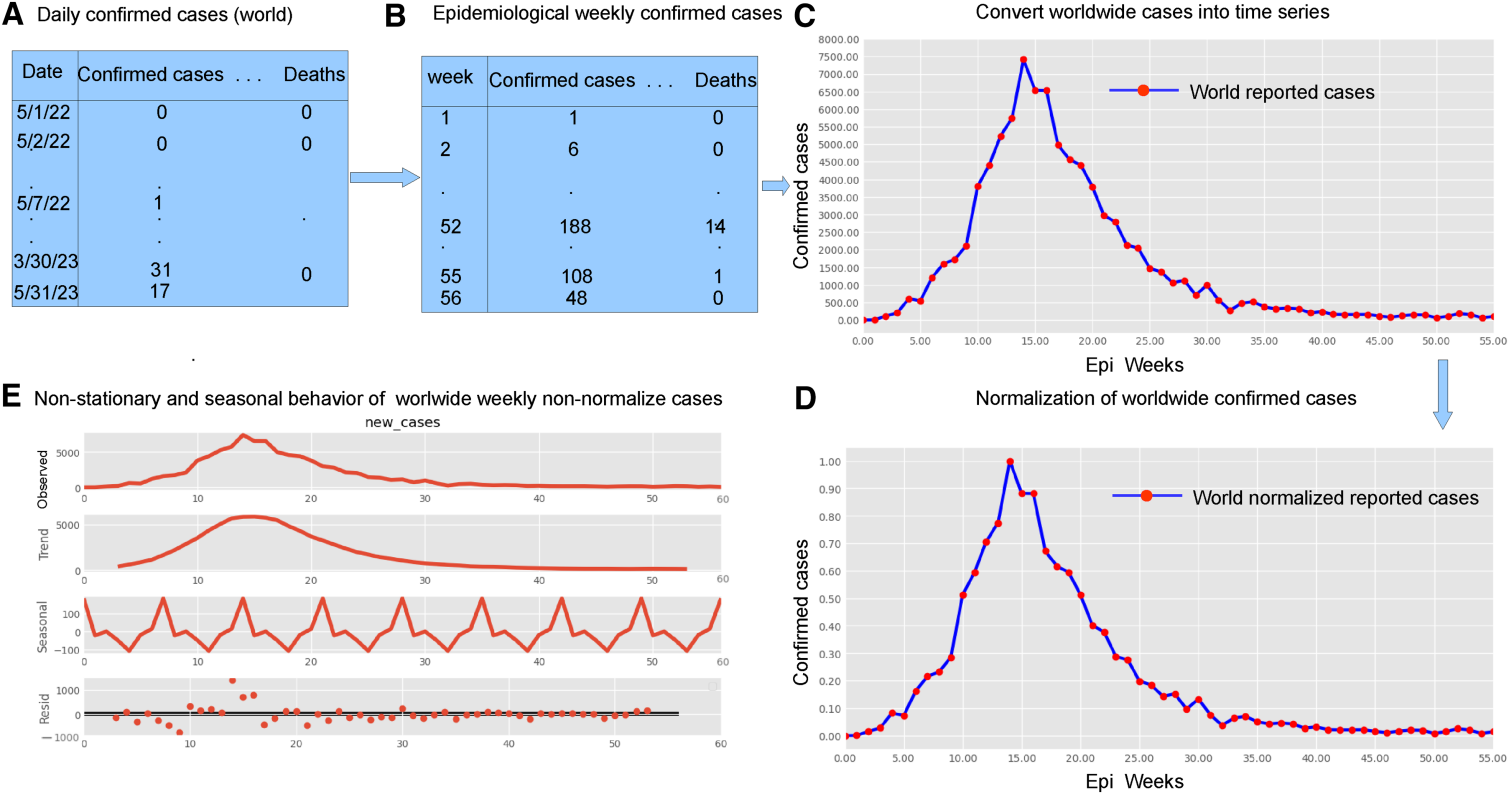
Preparing time series data: This figure shows five important phases: data pre-processing steps (panels **A** and **B**, top left), weekly time series of reported cases (panel **C**, top right), weekly time series of reported cases on a normalized scale (panel **D** bottom right), and the decomposition of the confirmed cases to check seasonality and stationarity (panel **E**, bottom left). Given the ADF Statistic of −2.04679, which is negative, and a p-value of 0.26644, exceeding the significance threshold of 0.05, we can infer that the EPI weekly worldwide data exhibits non-stationarity. Panel **E** also demonstrates the seasonality patterns, as the seasonal decomposition shows the rise and fall, with a period of seven. We observe an increasing trend in the number of reported cases until the EPI week of 14 (between July and August, during the summer), then started decreasing. Panel **E** displays the additive decomposition, where the seasonal chart scales between −100 to 100 and the trend chart scales between −1,000 to 1,000.

**Figure 3:**
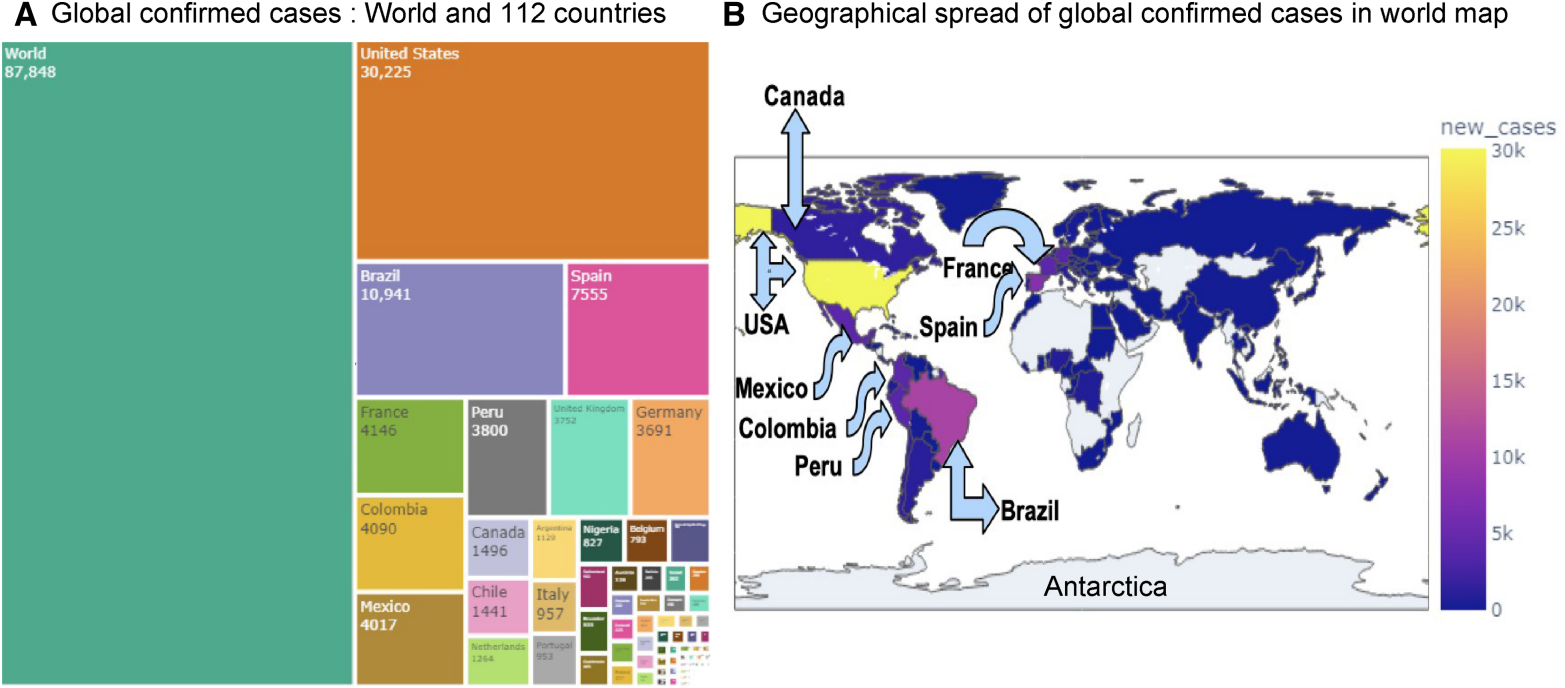
Visualization of globally reported Mpox cases: Panel (**A**) displays the confirmed Mpox cases across 112 countries using a treemap visualization. Panel (**B**) shows the global and regional spread of confirmed Mpox cases on a world map. Among all countries, the USA has recorded the highest number of cases (30,225), followed by Brazil (10,941), Spain (7,555), France (4,146), Colombia (4,090), and Mexico (4,017).

**Figure 4:**
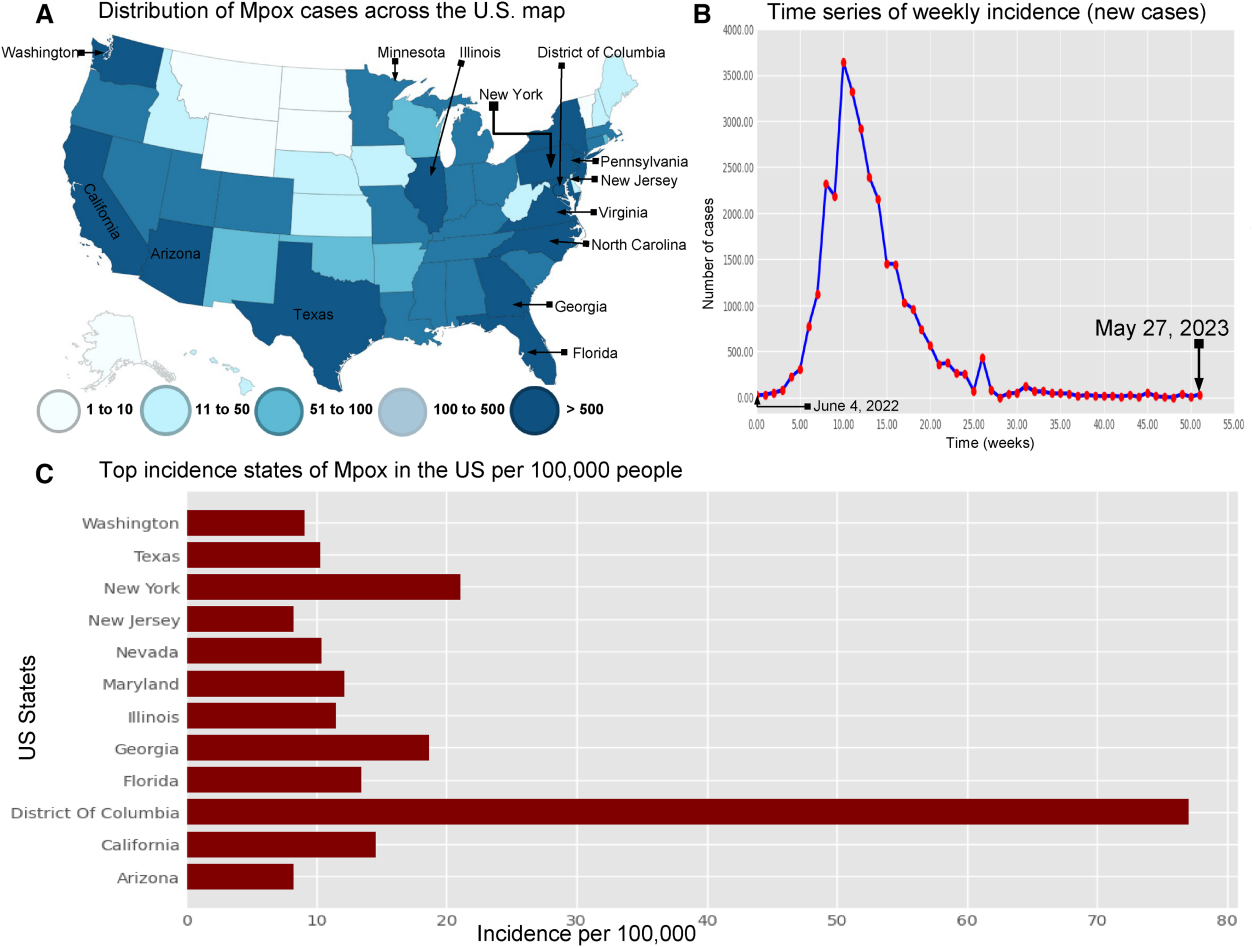
Visualization the spread of Mpox cases in the USA: Panel (**A**) displays the distribution of Mpox cases (left top) across the US map (35) . Panel (**B**) shows the time series of confirmed Mpox cases (right top) from week 0 to 51, covering the period from June 4, 2022, to May 27, 2023. Panel (**C**) illustrates the states with the highest incidence of Mpox per 100,000 (100 k) people in the United States. This panel also shows that the District of Columbia (77.01% per 100 k) has higher Mpox incidence from May 2022 to May 2023. Next, the other states such as New York (18.61% per 100 k), California (14.58% per 100 k), Florida (13.44% per 100 k), Maryland (12.1% per 100 k), Illinois (11.48% per 100 k), Nevada (10.04% per 100 k), and Texas (10.33% per 100 k) are the higher risk of an epidemic. Additionally, Washington (9.06% per 100 k), Arizona (8.28% per 100 k), and New Jersey (25.57% per 100 k) have a higher chance of an epidemic compared to the other states.

**Figure 5:**
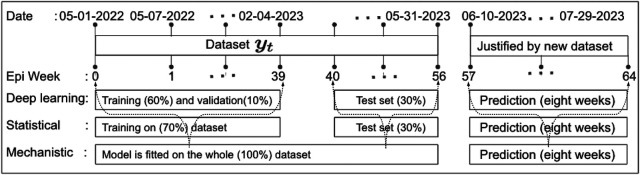
Data partitioning into training, validation, and testing sets: This figure illustrates the decomposition of the training, validation, and testing data based on the EPI week for deep learning (1D-CNN, LSTM, BiLSTM, hybrid CNN-LSTM, and CNN-BiLSTM), statistical (ARIMA and exponential smoothing), and deterministic models. Note that data from May 01 to May 31, 2023, was accessed on June 03, 2023. Later, new data for comparing model predictions were accessed on August 01, 2023.

The research materials and methods involve data pre-processing and building predictive models for time series data from May 1, 2022, to May 31, 2023. The first phase involves converting the daily dataset into a weekly time series, as shown in Figure 2, normalizing the data using Equation 1, visualizing mpox cases in global and US regions in Figures 3 and 4, respectively, and splitting the data into training and test sets, as depicted in Figure 5. The second phase involves optimizing and training the models using the best hyperparameters on a train set, which starts on week 0 (May 1, 2022) and ends on week 40 (February 11, 2023). Then, the trained models are evaluated on a test set from week 40 (February 11, 2023), to week 56 (May 31, 2023). Figures 2, 5 provide an overview of the entire procedure. Furthermore, a year is conventionally classified as an epidemic year for a given region if the incidence of Mpox exceeds 100 cases per 100,000 individuals in a given year, for example, a year between May 2022 and May 2023. A non-epidemic year when the incidence of Mpox remains below this threshold like Stolerman et al. (34). In this work, we use this threshold to identify which country has the epidemic year for Mpox and then use the model fitting using the deterministic model in some regions. From Figures 1, 4, we see no locations that may be considered epidemic years for Mpox disease, but the District of Columbia in the USA, is close to this threshold. Figure 3 visualizes global confirmed human Mpox cases by country, continent, and geographic region, and it reveals that Mpox is transmitted more in some regions of the world. It also reveals that the majority of cases are reported in the USA.

### 2.2 Processing the time series data

**Figure A1:**
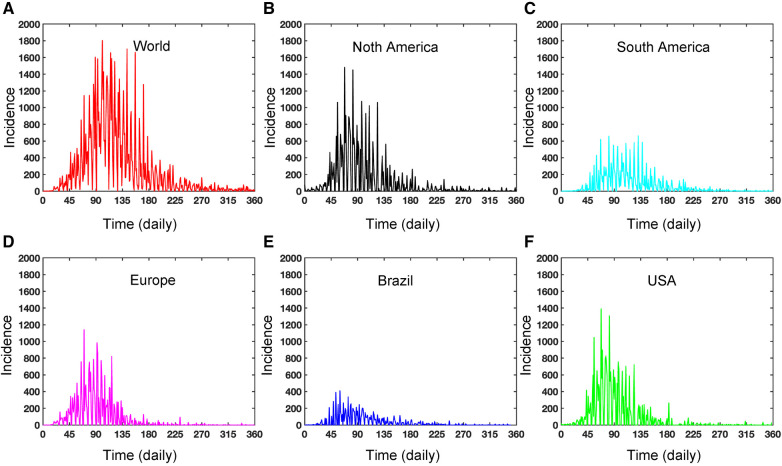
Dynamics of daily Mpox incidence: This figure illustrates the daily incidence of Mpox in various locations from May 2022 to May 2023. The figure exhibits seasonality and includes missing reported data. In contrast, the weekly data in Figure 2C captures the proper disease dynamics, which can be a pragmatic choice.

**Figure A2:**
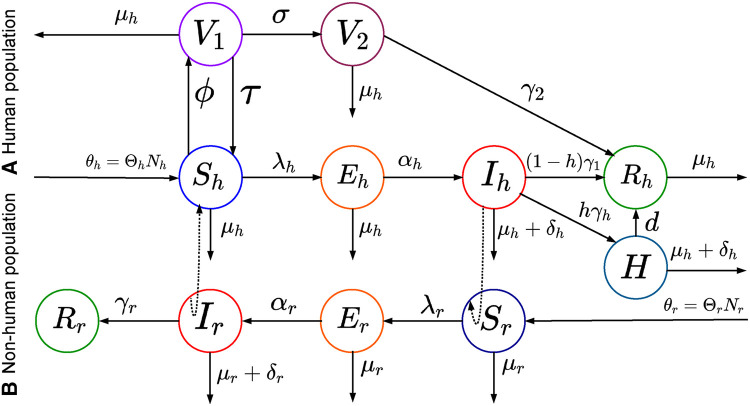
Schematic representation of the deterministic model: This figure shows a modified SEIR model with two-phase vaccinations (smallpox or dose one vaccine and dose two vaccines) for mpox virus transmissions.

Many real-world disease datasets exhibit temporal patterns, representing the number of infected cases at regular intervals, such as daily or weekly, naturally forming time-series data. Figures 2A,B and A1 (appendix) illustrate common issues stemming from insufficient or low reported data on weekends, particularly around Sundays. To mitigate the impact of reduced weekend reporting, we adopted the epidemiological week (EPI week) or CDC week (where each EPI week begins on Sunday and ends on Saturday) as depicted in Figure 2B. By filtering the data using the EPI week, as shown in Figure 2B, we aimed to reduce the noise inherent in the daily data shown in Figures 2A and A2. This resulted in grouping 395 data points into EPI weeks for each region, providing a dataset spanning 57 weeks.

#### 2.2.1 Data normalization

Modeling time series data presents challenges due to its dynamic and nonlinear nature, as highlighted by Tealab et al. (36) in 2017 and McFarland et al. (37) in 2018. Data normalization techniques, such as the Min-Max formula (Equation 1), are commonly used to address this issue. Notably, data normalization plays a crucial role in ensuring the quality of data prior to model training, and as such, it can significantly impact the performance of any subsequent analysis or modeling effort. The Min-Max formula (Equation 1) described by Wibawa et al. (38) in 2022 is used to normalize data in Figure 2D, resulting in smaller intervals within 0–1. Following (36, 37), we define the data normalization equation as

(1) $$y_{t}^{\mathrm{norm}}=\frac{y_{t}-y_{\min}}{y_{\max}-y_{\min}},$$

where $y_{t}^{\mathrm{norm}}$ is the result of normalization, and $y_{t}$ is the data to be normalized, while $y_{\min}$ and $y_{\max}$ stand for the minimum and maximum value of the data. The deterministic model, ARIMA, and Exponential smoothing models are independent of any other variable; hence, this study does not require normalization for these models. The only normalized data was used in the deep learning models such as CNN, LSTM, BiLSTM, and hybrid CNN-LSTM and CNN-BiLSTM, as normalization can help prevent vanishing and exploding gradients during training.

#### 2.2.2 Split time series data

We define the training, testing, and validation datasets. In 2018, Kuhn and Johnson (39) introduced a section titled “Data Splitting Recommendations,” and later in 2021 Nguyen et al. (40) outlined the limitations of using a sole “test set.” Of course, there are no fixed rules for separation training and testing data sets, nor is there an “ideal” ratio for splitting a dataset, but experts commonly used ratios such as 70:30; 80:20; 65:35; 60:40, etc. Given our current small dataset size, this experiment uses the first 60 percent of the data points in the time series as the training set, 10 percent as validation, and the last 30 percent of the data points as the “test set,” as depicted in Figure 5. Additionally, to validate the predictions made by the models, we consider another eight weeks of cases from June 2023 to July 2023 accessed this data on August 01, 2023.

## 3 Methods

**Figure 6:**
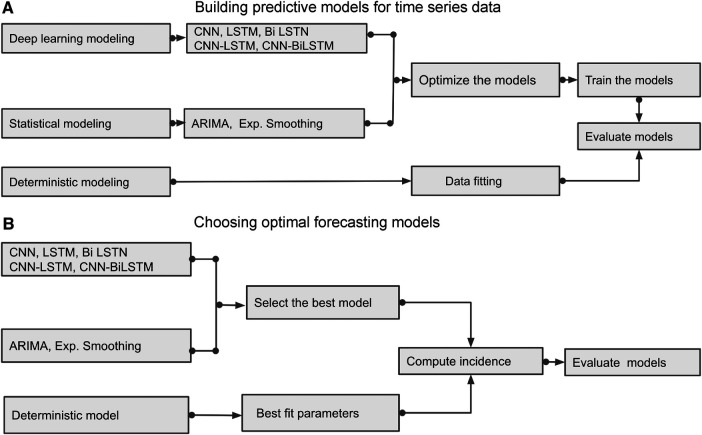
Building Blocks of deterministic and data-driven models: (**A**) displays different methods and demonstrates how to build the predictive models. (**B**) identifies the best predictive models by evaluating those models.

**Figure 7:**
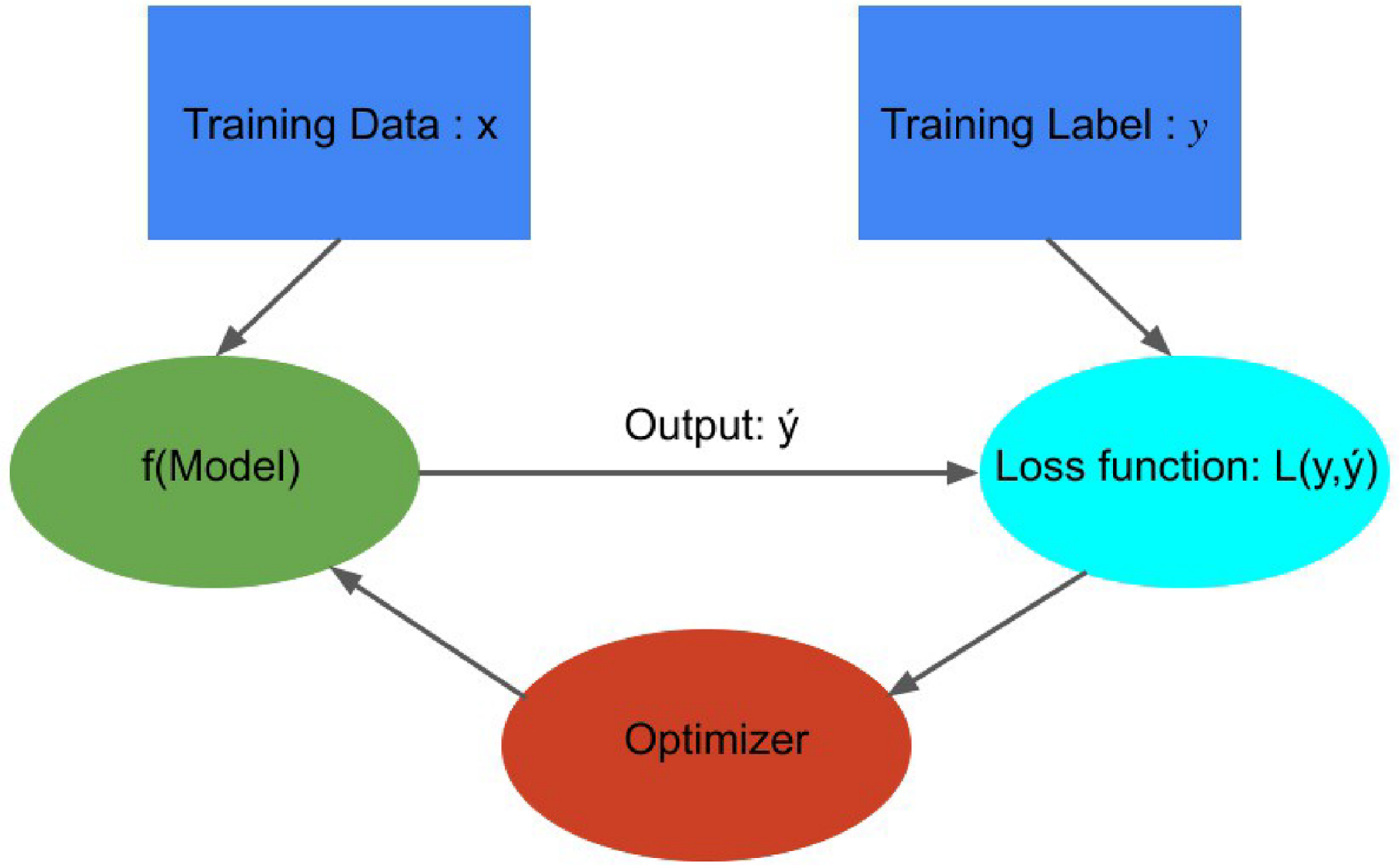
Building Blocks of data-driven models: This figure was adapted from Crick (41), illustrating how a model optimizer is generally used in model training. The loss function $L(y,\overset{´}{y})$ is computed by comparing predicted values $\left(\overset{´}{y}\right)$ and true values (y), and model parameters are adjusted to minimize $L(y,\overset{´}{y})$ that enhances the accuracy of prediction model f.

This section briefly outlines several time series prediction models: a deterministic model and various data-driven methods, including deep learning models such as CNN, LSTM, BiLSTM, hybrid CNN-LSTM, and CNN-BiLSTM along with two classical statistical models: ARIMA and exponential smoothing. We aim to demonstrate how to build and choose the optimal predictive models; Figures 6 and 7 show the building blocks of these methods used to analyze Mpox time series data.

### 3.1 Performance metrics

In this study, we used three performance criteria (42, 43), namely mean absolute error (MAE), root mean square error (RMSE), and normalized RMSE (nRMSE) to evaluate the predictive accuracy of the developed models, where lower values indicate better performance (44). To define these performance metrics, we set y as the actual data value, $\overset{´}{y}$ as the forecast value, and n as the number of observations. Then the mathematical formulas for these error evaluation measures are as follows:

(2) $$\text{MAE}=\frac{\sum_{t=1}^{n}\left|y-\overset{´}{y}\right|}{n},\;\text{RMSE}=\sqrt{\frac{\sum_{t=1}^{n}(y-\overset{´}{y}{)}^{2}}{n}},\;\text{and}\;\text{nRMSE}=\frac{\text{RMSE}}{\left(y_{\max}-y_{\min}\right)}.$$

One can also compute nRMSE based on the average $\left(\bar{y}\right)$ of the actual data values, calculated as $\frac{\text{RMSE}}{\bar{y}}$.

### 3.2 Deterministic model

We built a model to analyze the transmission of Mpox within and between human and rodent (non-human) populations. Extending the SEIR-like vector-borne models (24, 25), we introduce a deterministic model and then observe how monkeypox spreads through the human population over time by inputting different values for these variables and running simulations. The models are based on the following assumptions: (1) All individuals are identical, that is, age, sex, social status, and other characteristics do not affect the probability of infection; (2) Human population mixing is homogeneous; (3) There is no inherited immunity; (4) With these assumptions, we consider the population sizes N(t) with a per capita birth rate Θ in susceptible class and death rate μ in all classes. Also, θ=ΘN(t) represents the new birth in the susceptible class. Therefore, the rate of change of a population is proportional to the total population size, i.e. (Equation 3),

(3) $$\dot{N}(t)=\Theta N(t)-\mu N(t)=rN(t),\;\text{where}\;r=\Theta -\mu \;\text{is the population growth rate.}$$

We now propose a deterministic model on the transmission dynamics of monkeypox consisting of two groups: the human population and the rodent (non-human) population. The high-risk group will increase transmission in the low-risk group, and the low-risk group will decrease transmission in the high-risk group. These interactions can be represented using the following form (Equation 4):

(4) $$\lambda =\left(\begin{array}{c} \lambda_{h}\\ \lambda_{r} \end{array}\right)=\left(\begin{array}{cc} \beta_{hh} & \beta_{hr}\\ \beta_{rh} & \beta_{rr} \end{array}\right)\left(\begin{array}{c} I_{h}\\ I_{r} \end{array}\right)=\left(\begin{array}{c} \beta_{hh}I_{h}+\beta_{hr}I_{r}\\ \beta_{rh}I_{h}+\beta_{rr}I_{r} \end{array}\right),$$

where the group-specific force of infections, $\lambda =\left(\begin{array}{c} \lambda_{h}\\ \lambda_{r} \end{array}\right)$, the transmission matrix, $T=\left(\begin{array}{cc} \beta_{hh} & \beta_{hr}\\ \beta_{rh} & \beta_{rr} \end{array}\right)$, and the infectious class, $I=\left(\begin{array}{c} I_{h}\\ I_{r} \end{array}\right)$. The diagonal and off-diagonal of T represent within-group and between-group interactions, respectively, and we assume that det( T) ≥0, as it is reasonable to disease transmission. Additionally, the first component h of $\beta_{hr}$ indicates the infected group , and the second component r indicates the infectious group. In this model, each population can infect the other, but the infection moves through the populations separately. To model the spread of Mpox in a metropolitan area, Yuan et al. (24) suggest a transmission risk and control strategy for Mpox using a matrix of contracts among the groups. With that, we assume the transmission rates are greater within groups than between them, and $\beta_{hr}=\beta_{rh}=c_{0}(1-k_{1}),\beta_{hh}=c_{0}k_{1}$, and $\beta_{rr}=c_{0}k_{2}$, where $c_{0}$ the baseline contact rate among the overall population and $k_{1}$ and $k_{2}$ are the proportion of contacts within the human and rodent (non-human) overall contacts, respectively. Moreover, the current outbreak of Mpox suggests that sustained human-to-human transmission is quite feasible, and smallpox vaccination protects against Mpox. As of June 2023, similar to the smallpox vaccination (13), Mpox vaccines such as (45) JYNNEOS (46) and ACAM2000 (47) are recommended for individuals at higher risk during the ongoing Mpox outbreak (45). Therefore, incorporating the vaccination class V with a rate ϕ of susceptible populations into the models Peter et al. (48, 49) is required for the Mpox model, which has been proven effective in preventing Mpox infections. Moreover, the smallpox vaccine and dose-1 Mpox vaccination may not offer complete protection against Mpox; therefore, τ converts the vaccinated class back to a susceptible class. Additionally, besides the two groups, the human population is further subdivided into seven compartments: susceptible humans $S_{h}(t)$, exposed humans $E_{h}(t)$, infected humans $I_{h}(t)$, hospitalized humans H(t), smallpox or dose-1 Mpox vaccinated humans $V_{1}(t)$, dose-2 vaccinated humans $V_{2}(t)$, and recovered humans $R_{h}(t)$. The rodent population is also subdivided into three compartments: susceptible rodents $S_{r}(t)$, exposed rodents $E_{r}(t)$, infected rodents $I_{r}(t)$, and recovered rodents $R_{r}(t)$ (Table 1).

**Table 1:** Model’s parameters of Figure A2.

Parameters	Description
$N_{h}$	Total size of the human population
$N_{r}$	Total size of rodent population
$\Theta_{h}$	Human recruitment rate
$\Theta_{r}$	Rodent recruitment rate
λ	Group-specific force of infections
$\mu_{h}$	Natural death rate of human
$\mu_{r}$	Natural death rate of rodent
$\delta_{h}$	Weekly disease-induced death rate in the human populations
$\delta_{r}$	Weekly disease-induced death rate in the rodent populations
$\frac{1}{\alpha_{h}}$	Average incubation period of Mpox in the human population
$\frac{1}{\alpha_{r}}$	Average incubation period of Mpox in the rodent population
$\frac{1}{\gamma_{1}}$	Average number of weeks of recovery needed for infectious individuals
$\frac{1}{\gamma_{2}}$	Average number of weeks of recovery needed for dose-2 vaccinated individuals
h	Proportion of hospitalization
$\frac{1}{\gamma_{h}}$	Average number of weeks between symptom onsets and hospitalization
$\frac{1}{d}$	Average number of weeks of recovery needed for hospitalized individuals
$\frac{1}{\gamma_{r}}$	Average number of weeks of recovery needed for infectious rodent
ϕ	Smallpox or dose-1 vaccine rate
σ	Dose-2 vaccine rate
τ	Smallpox or dose-1 vaccine failure rate
$c_{0}$	Baseline contact rate among the overall population
$k_{1}$	Proportion of contacts within the human in overall contacts
$k_{2}$	Scaling factor of contact rate among the rodent compared to baseline contact

Employing the parameters listed in Table 1 and referring to the compartmental diagram (Figure A2) provided in Appendix A, we can derive the following system of differential equations:

(5) $$\begin{array}{r} \begin{array}{cc} Group\;A:\;Human\;population\\ & {\dot{S}}_{h}=\theta_{h}-\lambda_{h}\frac{S_{h}}{N_{h}}-(\mu_{h}+\phi )S_{h}+\tau V_{1}\;\\ & {\dot{E}}_{h}=\lambda_{h}\frac{S_{h}}{N_{h}}-(\mu_{h}+\alpha_{h})E_{h}\;\\ & {\dot{I}}_{h}=\alpha_{h}E_{h}-[(1-h)\gamma_{1}+h\gamma_{h}+(\mu_{h}+\delta_{h})]I_{h}\;\\ & \dot{H}=h\gamma_{h}I_{h}-(d+\mu_{h}+\delta_{h})H\;\\ & {\dot{V}}_{1}=\phi S_{h}-(\tau +\mu_{h}+\sigma )V_{1}\;\\ & {\dot{V}}_{2}=\sigma V_{1}-(\mu_{h}+\sigma_{2})V_{2}\;\\ & {\dot{R}}_{h}=\gamma_{2}V_{2}+(1-h)\gamma_{1}I_{h}+dH-\mu_{h}R_{h}\;\\ Group\;B:\;Rodent\;population\;\\ & {\dot{S}}_{r}=\theta_{r}-\lambda_{r}\frac{S_{r}}{N_{r}}-\mu_{r}S_{r}\;\\ & {\dot{E}}_{r}=\lambda_{r}\frac{S_{r}}{N_{r}}-(\mu_{r}+\alpha_{r})E_{r}\;\\ & {\dot{I}}_{r}=\alpha_{r}E_{r}-(\mu_{r}+\delta_{r}+\gamma_{r})I_{r}\;\\ & {\dot{R}}_{r}=\gamma_{r}I_{r}-\mu_{r}R_{r}\; \end{array} \end{array}$$

with the initial conditions in a biologically feasible region $\Gamma_{h}\times \Gamma_{r}$, where $\Gamma_{h}\;\mathrm{and}\;\Gamma_{r}$ are defined by the Equation 6 below:

(6) $$\begin{array}{cc} & \Gamma_{h}=\{(S_{h},E_{h},I_{h},H,V_{1},V_{2},R_{h})\in R_{+}^{7}\;:\;S_{h}+E_{h}+I_{h}+H+V_{1}+V_{2}+R_{h}\leq N_{h}\}\;\text{and}\\ & \Gamma_{r}=\{(S_{r},E_{r},I_{r},R_{r})\in R_{+}^{4}\;:\;S_{r}+E_{r}+I_{r}+R_{r}\leq N_{r}\}. \end{array}$$

#### 3.2.1 Model analysis

By summing the first seven equations of group A in the (Equation 5), we have ${\dot{S}}_{h}+{\dot{E}}_{h}+{\dot{I}}_{h}+\dot{H}+{\dot{V}}_{1}+{\dot{V}}_{2}+{\dot{R}}_{h}=\theta_{h}-\mu_{h}(S_{h}+E_{h}+I_{h}+H+$ $V_{1}+V_{2}+R_{h})-\delta_{h}(I_{h}+H)=\theta_{h}-\mu_{h}N_{h}-\delta_{h}(I_{h}+H)$. Using $\theta_{h}=\Theta_{h}N_{h}$, the differential equation for the human population $N_{h}$ is given as follows:

(7) $${\dot{N}}_{h}=(\Theta_{h}-\mu_{h})N_{h}-\delta_{h}(I_{h}+H)$$

Moreover, Figure 12 illustrates the significance of vaccination for the US population. Similarly, for the non-human population, $N_{r}$, we have ${\dot{S}}_{r}+{\dot{E}}_{r}+{\dot{I}}_{r}+{\dot{R}}_{r}=\theta_{r}-\mu_{r}(S_{r}+E_{r}+I_{r}+R_{r})-\delta_{r}I_{r}=\theta_{r}-\mu_{r}N_{r}-\delta_{r}I_{r}$, and using $\theta_{r}=\Theta_{r}N_{r}$ the corresponding differential is given as follows:

(8) $${\dot{N}}_{r}=(\Theta_{r}-\mu_{r})N_{r}-\delta_{r}I_{r}.$$

Here, $N_{h}$ and $N_{r}$ are sums of state variables for human and rodent populations, respectively, which are not constants in general. However, they are constants when $\Theta_{h}=\mu_{h},\delta_{h}=0$, and $\Theta_{r}=\mu_{r},\delta_{r}=0$ as ${\dot{N}}_{h}=0$, ${\dot{N}}_{r}=0$, respectively (Equations 7–8).

#### 3.2.2 Equilibrium state

There are two types of equilibrium points for the model given in (Equation 5). One is the Disease-Free Equilibrium(DFE) or Mpox-Free equilibrium point, $\epsilon^{0}=(S_{h}^{0},E_{h}^{0},I_{h}^{0},H^{0},V_{1}^{0},V_{2}^{0},R_{h}^{0},S_{r}^{0},E_{r}^{0},I_{r}^{0},R_{r}^{0})=(\frac{\theta_{h}(\mu_{h}+\sigma +\tau )}{\mu_{h}(\mu_{h}+\sigma +\tau )+\phi (\mu_{h}+\sigma ))},0,0,0,\frac{\theta_{h}\phi }{\mu_{h}(\mu_{h}+\sigma +\tau )+\phi (\mu_{h}+\sigma )},\frac{\theta_{h}\sigma \phi }{\left(\gamma_{2}+\mu_{h})(\mu_{h}(\mu_{h}+\sigma +\tau )+\phi (\mu_{h}+\sigma )\right)},\frac{\gamma_{2}\theta_{h}\sigma \phi }{\mu_{h}(\gamma_{2}+\mu_{h})(\mu_{h}(\mu_{h}+\sigma +\tau )+\phi (\mu_{h}+\sigma ))},\frac{\theta_{r}}{\mu_{r}},0,0,0)$, which corresponds to the absence of infectious individuals. The second equilibrium point is called endemic equilibrium, represented by $\left(S_{h}^{*},E_{h}^{*},I_{h}^{*},H^{*},V_{1}^{*},V_{2}^{*},R_{h}^{*},S_{r}^{*},E_{r}^{*},I_{r}^{*}\right)$, which can be obtained by equating the right-hand side of the system (Equation 5) with zero. We use the symbolic packages of the software Mathematica for the analytic computations in our deterministic model. Using the notation $T_{1}=\mu_{h}+\alpha_{h},T_{2}=(1-h)\gamma_{1},T_{3}=\mu_{h}+\delta_{h},T_{4}=\tau +\mu_{h}+\sigma ,T_{5}=\mu_{r}+\alpha_{r},T_{6}=\mu_{r}+\delta_{r}$, and $T_{7}=\mu_{h}+\sigma$ in the system (Equation 5), we obtain the endemic equilibrium point as follows:

$$\begin{array}{rl} & S_{h}^{*}=\frac{N_{h}T_{4}\theta_{h}}{T_{4}(\lambda_{h}+N_{h}(\mu_{h}+\phi ))-N_{h}\tau \phi },E_{h}^{*}=\frac{T_{4}\theta_{h}\lambda_{h}}{T_{1}T_{4}(\lambda_{h}+N_{h}\mu_{h})+N_{h}T_{1}(T_{4}-\tau )\phi }\\ & I_{h}^{*}=\frac{T_{4}\alpha_{h}\theta_{h}\lambda_{h}}{T_{1}(h*\gamma_{h}+T_{2}+T_{3})(T_{4}(\lambda_{h}+N_{h}(\mu_{h}+\phi ))-N_{h}\tau \phi )},H^{*}=\frac{h\gamma_{h}T_{4}\alpha_{h}\theta_{h}\lambda_{h}}{T_{1}(d+T_{3})(h\gamma_{h}+T_{2}+T_{3})(T_{4}(\lambda_{h}+N_{h}(\mu_{h}+\phi ))-N_{h}\tau \phi )}\\ & V_{1}^{*}=\frac{N_{h}\theta_{h}\phi }{T_{4}(\lambda_{h}+N_{h}(\mu_{h}+\phi ))-N_{h}\tau \phi },V_{2}^{*}=\frac{N_{h}\theta_{h}\sigma \phi }{\left(\gamma_{2}+\mu_{h})(T_{4}(\lambda_{h}+N_{h}(\mu_{h}+\phi ))-N_{h}\tau \phi \right)}\\ & R_{h}^{*}=\frac{(d(h\gamma_{h}+T_{2})+T_{2}T_{3})T_{4}\alpha_{h}\theta_{h}\lambda_{h}(\gamma_{2}+\mu_{h})+N_{h}T_{1}(d+T_{3})(h\gamma_{h}+T_{2}+T_{3})\gamma_{2}\theta_{h}\sigma \phi }{T_{1}(d+T_{3})(h\gamma_{h}+T_{2}+T_{3})\mu_{h}(\gamma_{2}+\mu_{h})(T_{4}(\lambda_{h}+N_{h}(\mu_{h}+\phi ))-N_{h}\tau \phi )}\\ & S_{r}^{*}=\frac{N_{r}\theta_{r}}{\lambda_{r}+N_{r}\mu_{r}},E_{r}^{*}=\frac{\theta_{r}\lambda_{r}}{T_{5}\lambda_{r}+N_{r}T_{5}\mu_{r}},I_{r}^{*}=\frac{\alpha_{r}\theta_{r}\lambda_{r}}{T_{5}(T_{6}+\gamma_{r})(\lambda_{r}+N_{r}\mu_{r})},R_{r}^{*}=\frac{\alpha_{r}\gamma_{r}\theta_{r}\lambda_{r}}{T_{5}(T_{6}+\gamma_{r})(\lambda_{r}+N_{r}\mu_{r})}. \end{array}$$

#### 3.2.3 Basic reproduction number

In our proposed Mpox model (Equation 5), compartments $S_{h},V_{1},V_{2},R_{h}$, and $S_{r}$ represent the disease-free states , while compartments $E_{h},I_{h},H,E_{r}$, and $I_{r}$ represent the disease class. To investigate whether an infected individual will promote an epidemic outbreak in a susceptible population, we analyze the number of secondary infections produced by an infected individual during the transmission period, called the *basic reproduction number* $R_{0}$, at the DFE $\epsilon^{0}$. Using the technique (50, 51), the next-generation matrix (NGM), $\kappa =FV^{-1}$ at the DFE is defined as

(9) $$\kappa =\left(\begin{array}{ccccc} \frac{\alpha_{h}\beta_{hh}\theta_{h}T_{4}}{N_{h}T_{1}(h\gamma_{h}+T_{2}+T_{3})(\mu_{h}T_{4}+\phi T_{7})} & \frac{\beta_{hh}\theta_{h}T_{4}}{N_{h}(h\gamma_{h}+T_{2}+T_{3})(muh\text{T4}+\text{T7}\phi )} & 0 & \frac{\alpha_{r}\beta_{hr}\theta_{h}T_{4}}{N_{h}T_{5}T_{6}(\mu_{h}T_{4}+\phi T_{7})} & \frac{\beta_{hr}\theta_{h}T_{4}}{N_{h}T_{6}(\mu_{h}T_{4}+\phi T_{7})}\\ 0 & 0 & 0 & 0 & 0\\ 0 & 0 & 0 & 0 & 0\\ \frac{\alpha_{h}\beta_{rh}\theta_{r}}{\mu_{r}N_{r}T_{1}(h\gamma_{h}+T_{2}+T_{3})} & \frac{\beta_{rh}\theta_{r}}{\mu_{r}N_{r}(h\gamma_{h}+T_{2}+T_{3})} & 0 & \frac{\alpha_{r}\beta_{rr}\theta_{r}}{\mu_{r}N_{r}T_{5}T_{6}} & \frac{\beta_{rr}\theta_{r}}{\mu_{r}N_{r}T_{6}}\\ 0 & 0 & 0 & 0 & 0 \end{array}\right),$$

where the notations $T_{1}=\mu_{h}+\alpha_{h},T_{2}=(1-h)\gamma_{1},T_{3}=\mu_{h}+\delta_{h},T_{4}=\tau +\mu_{h}+\sigma ,T_{5}=\mu_{r}+\alpha_{r},T_{6}=\mu_{r}+\delta_{r}$, and $T_{7}=\mu_{h}+\sigma$ are used in the matrices

$$\begin{array}{rl} & F=\left(\begin{array}{ccccc} 0 & \frac{\beta \text{hh}\theta \text{h}(\mu \text{h}+\sigma +\tau )}{\text{Nh}(\mu \text{h}(\mu \text{h}+\sigma +\tau )+\phi (\mu \text{h}+\sigma ))} & 0 & 0 & \frac{\beta \text{hr}\theta \text{h}(\mu \text{h}+\sigma +\tau )}{\text{Nh}(\mu \text{h}(\mu \text{h}+\sigma +\tau )+\phi (\mu \text{h}+\sigma ))}\\ 0 & 0 & 0 & 0 & 0\\ 0 & 0 & 0 & 0 & 0\\ 0 & \frac{\beta \text{rh}\theta \text{r}}{\mu \text{r}\text{Nr}} & 0 & 0 & \frac{\beta \text{rr}\theta \text{r}}{\mu \text{r}\text{Nr}}\\ 0 & 0 & 0 & 0 & 0 \end{array}\right),\\ & V=\left(\begin{array}{ccccc} \alpha \text{h}+\mu \text{h} & 0 & 0 & 0 & 0\\ -\alpha_{h} & \delta_{h}+\gamma_{1}(1-h)+h\gamma_{h}+\mu_{h} & 0 & 0 & 0\\ 0 & -h\gamma_{h} & d+\delta_{h}+\mu_{h} & 0 & 0\\ 0 & 0 & 0 & \alpha_{r}+\mu_{r} & 0\\ 0 & 0 & 0 & -\alpha_{r} & \delta_{r}+\mu_{r} \end{array}\right),\;\text{and}\\ & V^{-1}=\left(\begin{array}{ccccc} \frac{1}{\alpha_{h}+\mu_{h}} & 0 & 0 & 0 & 0\\ \frac{\alpha_{h}}{\left(\alpha_{h}+\mu_{h})(\delta_{h}+\gamma_{1}(1-h)+h\gamma_{h}+\mu_{h}\right)} & \frac{1}{\delta_{h}+\gamma_{1}(1-h)+h\gamma_{h}+\mu_{h}} & 0 & 0 & 0\\ \frac{h\alpha_{h}\gamma_{h}}{\left(\alpha_{h}+\mu_{h})(d+\delta_{h}+\mu_{h})(\delta_{h}+\gamma_{1}(1-h)+h\gamma_{h}+\mu_{h}\right)} & \frac{h\gamma_{h}}{\left((d+\delta_{h}+\mu_{h})(\delta_{h}+\gamma_{1}(1-h)+h\gamma_{h}+\mu_{h}\right)} & \frac{1}{d+\delta_{h}+\mu_{h}} & 0 & 0\\ 0 & 0 & 0 & \frac{1}{\alpha_{r}+\mu_{r}} & 0\\ 0 & 0 & 0 & \frac{\alpha_{r}}{\left(\alpha_{r}+\mu_{r})(\delta_{r}+\mu_{r}\right)} & \frac{1}{\delta_{r}+\mu_{r}} \end{array}\right). \end{array}$$

The NGM (Equation 9) is a non-negative matrix, and, as such, we expect that there will be a single, unique eigenvalue that is positive, real, and strictly greater than all the others. The corresponding characteristic equation of Equation 9 is det(κ−λI)=0, and this gives the eigenvalues (Equation 10)

(10) $$\lambda_{1}=0,0,0,\lambda_{2}=\frac{B_{1}-\sqrt{B_{2}+B_{3}^{2}}}{B_{4}},\lambda_{3}=\frac{B_{1}+\sqrt{B_{2}+B_{3}^{2}}}{B_{4}},$$

where, $B_{1}\;:\;=(\alpha_{r}\beta_{rr}\theta_{r}N_{h}T_{1}(h\gamma_{h}+T_{2}+T_{3})(\phi (\mu_{h}+\sigma )+\mu_{h}T_{4})+\alpha_{h}\beta_{hh}\theta_{h}\mu_{r}N_{r}T_{4}T_{5}T_{6}$,

$B_{2}=4\alpha_{h}\alpha_{r}\theta_{h}\theta_{r}\mu_{r}N_{h}N_{r}T_{1}T_{4}T_{5}T_{6}(h\gamma_{h}+T_{2}+T_{3})(\beta_{hr}\beta_{rh}-\beta_{hh}\beta_{rr})(\phi (\mu_{h}+\sigma )+\mu_{h}T_{4})$,

$B_{3}=(\alpha_{h}\beta_{rr}\theta_{r}N_{h}T_{1}(\gamma_{h}h+T_{2}+T_{3})(\phi (\mu_{h}+\sigma )+\mu_{h}T_{4})+\alpha_{h}\beta_{hh}\theta_{h}\mu_{r}N_{r}T_{4}T_{5}T_{6})$, and

$B_{4}=(2\mu_{r}N_{h}N_{r}T_{1}T_{5}T_{6}(h\gamma_{h}+T_{2}+T_{3})(\phi (\mu_{h}+\sigma )+\mu_{h}T_{4})$.

Since det(T)≥0, $B_{2}+B_{3}^{2}$ is well defined and positive, that implies $\lambda_{3}\geq \lambda_{2}$. Now, the basic reproduction number is defined as the largest eigenvalue (spectral radius) of the NGM (Equation 9), and it can be obtained as follows:

(11) $$R_{0}=\frac{B_{1}+\sqrt{B_{2}+B_{3}^{2}}}{B_{4}}.$$

Remark 1. From an epidemiological perspective, an epidemic outbreak will occur if and only if $R_{0}>1$. Moreover if det(T)=0, then $B_{2}=0$, and the new basic reproduction number becomes

(12) $$R_{0}=\frac{B_{1}+B_{3}}{B_{4}}=\frac{\alpha_{h}\beta_{hh}\theta_{h}T_{4}}{N_{h}T_{1}(\gamma_{h}h+T_{2}+T_{3})(\phi (\mu_{h}+\sigma )+\mu_{h}T_{4})}+\frac{\alpha_{r}\beta_{rr}\theta_{r}}{\mu_{r}N_{r}T_{5}T_{6}}.$$

By substituting the values of $T_{i}$ for i=1,…,6, $\theta_{h}=\Theta_{h}N_{h}$, and $\theta_{r}=\Theta_{r}N_{r}$ into (Equation 12), we derive the resulting

(13) $$R_{0}=\frac{\alpha_{h}\beta_{hh}\Theta_{h}(\mu_{h}+\sigma +\tau )}{\left(\alpha_{h}+\mu_{h})(\mu_{h}(\mu_{h}+\sigma +\tau )+\phi (\mu_{h}+\sigma ))(\delta_{h}+\gamma_{1}(1-h)+\gamma_{h}h+\mu_{h}\right)}+\frac{\alpha_{r}\beta_{rr}\Theta_{r}}{\mu_{r}(\alpha_{r}+\mu_{r})(\delta_{r}+\mu_{r})}.$$

Remark 2. Increasing the vaccination rate ϕ among susceptible individuals $S_{h}$ leads to an increase in $B_{4}$. This increase in vaccination is particularly significant as it results in a reduction of $R_{0}$, which plays a pivotal role in effectively controlling the transmission of the disease.

#### 3.2.4 Sensitivity analysis of basic reproduction $R_{0}$

As epidemiological models often involve estimated or fitted parameters, the inherent uncertainty lies in the values of these parameters when making conclusions about the underlying epidemic. Therefore, we carried out a sensitivity analysis of this model parameter concerning the basic reproduction number. The normalized sensitivity index of $R_{0}$ used in Ngungu et al. (52) and Samuel et al. (53) with respect to parameter p is given by

(14) $$S_{\;p}^{R_{0}}=\frac{\;p}{R_{0}}\frac{\partial R_{0}}{\partial p}.$$

When $S_{\;p}^{R_{0}}$ is positive for the parameter p, it indicates an increase in $R_{0}$, while a negative value of $S_{\;p}^{R_{0}}$ for the parameter p suggests a decrease in $R_{0}$. Due to complexity of the actual $R_{0}$ (Equation 11), we consider the normalized sensitivity index (Equation 14) of $R_{0}$ (Equation 13) with respect to the parameter $p=(\beta_{hh},\alpha_{h},\Theta_{h},\mu_{h},\delta_{h},h,\gamma_{h},\gamma_{1},\sigma ,\tau ,\phi ,\beta_{rr},\alpha_{r},\Theta_{r},\mu_{r},\delta_{r})$ as follows:

$$\begin{array}{rl} & S_{\alpha_{h}}^{R_{0}}=\frac{\alpha_{h}}{R_{0}}\frac{\beta_{hh}\Theta_{h}(\mu_{h}+\sigma +\tau )}{\left(\alpha_{h}+\mu_{h})(\mu_{h}(\mu_{h}+\sigma +\tau )+\phi (\mu_{h}+\sigma ))(\gamma_{1}+\delta_{h}-h\gamma_{1}+\gamma_{h}h+\mu_{h}\right)}\\ & S_{\beta_{hh}}^{R_{0}}=\frac{\beta_{hh}}{R_{0}}\frac{\alpha_{h}\Theta_{h}(\mu_{h}+\sigma +\tau )}{\left(\alpha_{h}+\mu_{h})(\mu_{h}(\mu_{h}+\sigma +\tau )+\phi (\mu_{h}+\sigma ))(\gamma_{1}+\delta_{h}-h\gamma_{1}+\gamma_{h}h+\mu_{h}\right)}\\ & S_{\Theta_{h}}^{R_{0}}=\frac{\Theta_{h}}{R_{0}}\frac{\alpha_{h}\beta_{hh}(\mu_{h}+\sigma +\tau )}{\left(\alpha_{h}+\mu_{h})(\mu_{h}(\mu_{h}+\sigma +\tau )+\phi (\mu_{h}+\sigma ))(\gamma_{1}+\delta_{h}-h\gamma_{1}+\gamma_{h}h+\mu_{h}\right)}\\ & S_{\mu_{h}}^{R_{0}}=\frac{\mu_{h}}{R_{0}}\frac{A}{(\alpha_{h}+\mu_{h}{)}^{2}(\mu_{h}(\mu_{h}+\sigma +\tau )+\phi (\mu_{h}+\sigma ){)}^{2}(\gamma_{1}+\delta_{h}-h\gamma_{1}+h\gamma_{h}+\mu_{h}{)}^{2}}\\ & S_{\delta_{h}}^{R_{0}}=-\frac{\delta_{h}}{R_{0}}\frac{\alpha_{h}\beta_{hh}\Theta_{h}(\mu_{h}+\sigma +\tau )}{(\alpha_{h}+\mu_{h})(\mu_{h}(\mu_{h}+\sigma +\tau )+\phi (\mu_{h}+\sigma ))(\gamma_{1}+\delta_{h}-h\gamma_{1}+\gamma_{h}+\mu_{h}{)}^{2}}\\ & S_{h}^{R_{0}}=\frac{h}{R_{0}}\frac{\alpha_{h}\beta_{hh}\theta_{h}(\gamma_{1}-\gamma_{h})(\mu_{h}+\sigma +\tau )}{N_{h}(\alpha_{h}+\mu_{h})(\mu_{h}(\mu_{h}+\sigma +\tau )+\phi (\mu_{h}+\sigma ))(\gamma_{1}+\delta_{h}-h\gamma_{1}+h\gamma_{h}+\mu_{h}{)}^{2}}\\ & S_{\gamma_{h}}^{R_{0}}=-\frac{\gamma_{h}}{R_{0}}\frac{\alpha_{h}\beta_{hh}h\Theta_{h}(\mu_{h}+\sigma +\tau )}{(\alpha_{h}+\mu_{h})(\mu_{h}(\mu_{h}+\sigma +\tau )+\phi (\mu_{h}+\sigma ))(\gamma_{1}+\delta_{h}-h\gamma_{1}+h\gamma_{h}+\mu_{h}{)}^{2}}\\ & S_{\gamma_{1}}^{R_{0}}=-\frac{\gamma_{1}}{R_{0}}\frac{\alpha_{h}\beta_{hh}(1-h)\Theta_{h}(\mu_{h}+\sigma +\tau )}{(\alpha_{h}+\mu_{h})(\mu_{h}(\mu_{h}+\sigma +\tau )+\phi (\mu_{h}+\sigma ))(\gamma_{1}+\delta_{h}-h\gamma_{1}+h\gamma_{h}+\mu_{h}{)}^{2}}\\ & S_{\sigma }^{R_{0}}=-\frac{\delta_{h}}{R_{0}}\frac{\alpha_{h}\beta_{hh}\Theta_{h}\tau \phi }{\left(\alpha_{h}+\mu_{h})(\mu_{h}(\mu_{h}+\sigma +\tau )+\phi (\mu_{h}+\sigma ){)}^{2}(\gamma_{1}+\delta_{h}-h\gamma_{1}+h\gamma_{h}+\mu_{h}\right)}\\ & S_{\tau }^{R_{0}}=\frac{\tau }{R_{0}}\frac{\alpha_{h}\beta_{hh}\Theta_{h}\phi (\mu_{h}+\sigma )}{\left(\alpha_{h}+\mu_{h})(\mu_{h}(\mu_{h}+\sigma +\tau )+\phi (\mu_{h}+\sigma ){)}^{2}(\gamma_{1}+\delta_{h}-h\gamma_{1}+h\gamma_{h}+\mu_{h}\right)}\\ & S_{\phi }^{R_{0}}=-\frac{\phi }{R_{0}}\frac{\alpha_{h}\beta_{hh}\Theta_{h}(\mu_{h}+\sigma )(\mu_{h}+\sigma +\tau )}{\left(\alpha_{h}+\mu_{h})(\mu_{h}(\mu_{h}+\sigma +\tau )+\phi (\mu_{h}+\sigma ){)}^{2}(\gamma_{1}+\delta_{h}-h\gamma_{1}+h\gamma_{h}+\mu_{h}\right)}\\ & S_{\beta_{rr}}^{R_{0}}=\frac{\beta_{rr}}{R_{0}}\frac{\alpha_{r}\Theta_{r}}{\mu_{r}(\alpha_{r}+\mu_{r})(\delta_{r}+\mu_{r})}\\ & S_{\alpha_{r}}^{R_{0}}=\frac{\alpha_{r}}{R_{0}}\frac{\beta_{rr}\theta_{r}}{N_{r}(\alpha_{r}+\mu_{r}{)}^{2}(\delta_{r}+\mu_{r})}\\ & S_{\Theta_{r}}^{R_{0}}=\frac{\Theta_{r}}{R_{0}}\frac{\alpha_{r}\beta_{rr}}{\mu_{r}(\alpha_{r}+\mu_{r})(\delta_{r}+\mu_{r})}\\ & S_{\mu_{r}}^{R_{0}}=-\frac{\mu_{r}}{R_{0}}\frac{\alpha_{r}\beta_{rr}\Theta_{r}\left(2\mu_{r}(\alpha_{r}+\delta_{r})+\alpha_{r}\delta_{r}+3\mu_{r}^{2}\right)}{\mu_{r}^{2}(\alpha_{r}+\mu -r{)}^{2}(\delta_{r}+\mu_{r}{)}^{2}}\\ & S_{\delta_{r}}^{R_{0}}=-\frac{\delta_{r}}{R_{0}}\frac{\alpha_{r}\beta_{rr}\Theta_{r}}{\mu_{r}(\alpha_{r}+\mu_{r})(\delta_{r}+\mu_{r}{)}^{2}}\\ & A=\alpha_{h}\beta_{hh}\Theta_{h}(-(\alpha_{h}+\mu_{h})(\mu_{h}+\sigma +\tau )(\mu_{h}(\mu_{h}+\sigma +\tau )+\phi (\mu_{h}+\sigma ))\\ & \;-(\alpha_{h}+\mu_{h})(\mu_{h}+\sigma +\tau )(2\mu_{h}+\sigma +\tau +\phi )(\gamma_{1}+\delta_{h}-h\gamma_{1}+\gamma_{h}h+\mu_{h})\\ & \;+(\alpha_{h}+\mu_{h})(\mu_{h}(\mu_{h}+\sigma +\tau )+\phi (\mu_{h}+\sigma ))(\gamma_{1}+\delta_{h}-h\gamma_{1}+h\gamma_{h}+\mu_{h})\\ & \;-(\mu_{h}+\sigma +\tau )(\mu_{h}(\mu_{h}+\sigma +\tau )+\phi (\mu_{h}+\sigma ))(\gamma_{1}+\delta_{h}-h\gamma_{1}+h\gamma_{h}+\mu_{h})) \end{array}$$

**Figure 8:**
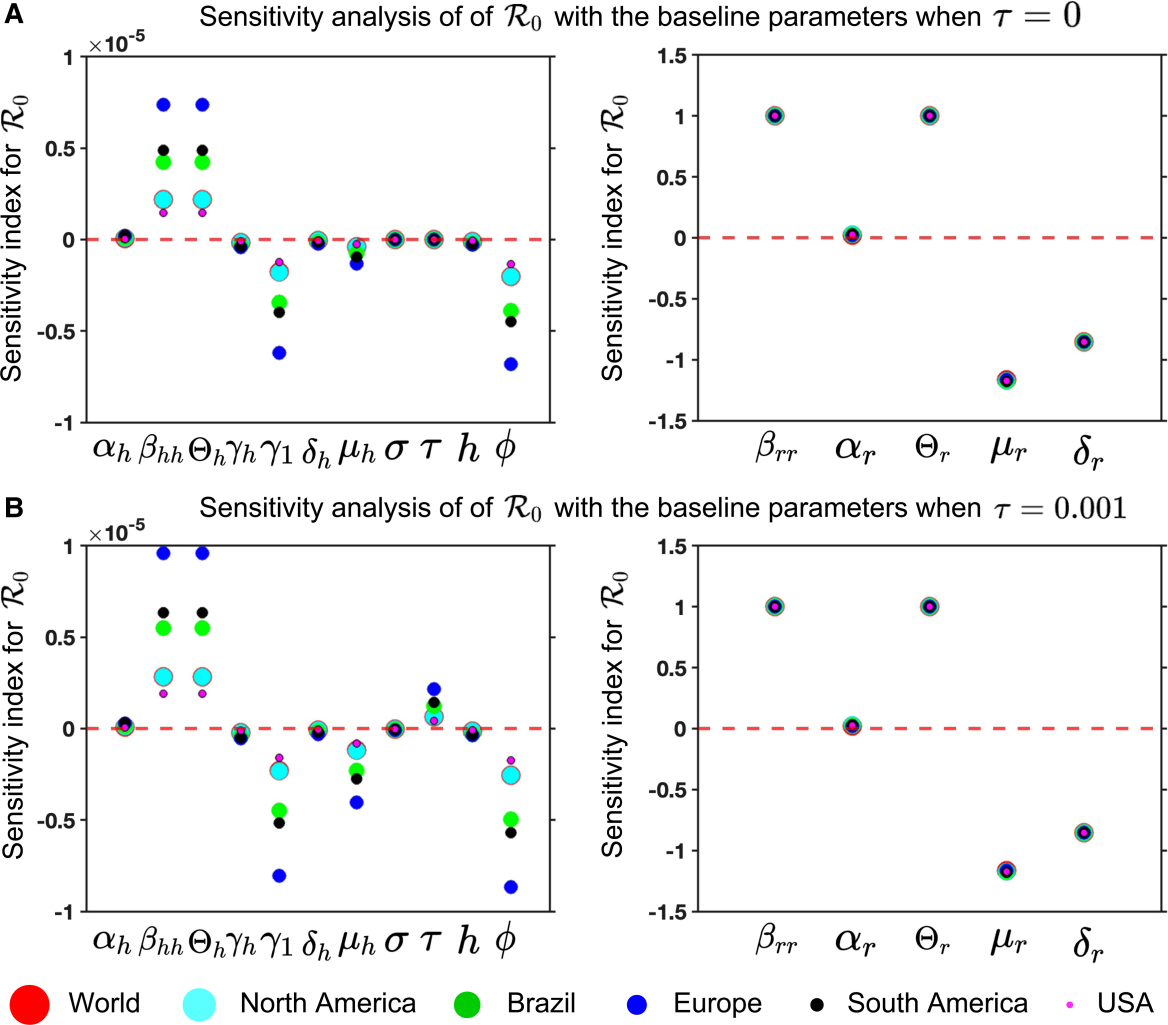
Mpox model sensitivities to its associated parameters for six different locations: Panel (**A**) and (**B**) illustrate the sensitivity index of $R_{0}$ (Equation 13) for τ=0 and τ=0.01, respectively. These are computed with the fitted parameters described in Table 3, variable parameters listed in Table 4, and remaining parameters given in Table 2. The figure demonstrates that the sensitivity index of the parameters $\beta_{hh},\theta_{h},\gamma_{1},\mu_{h},\tau$, and ϕ changes when the parameter values change, while the remaining parameters remain unchanged. In other words, the parameters $\alpha_{h},\gamma_{h},\delta_{h},\sigma$, and h are almost constant and do not depend on another parameter, but the remaining parameters show the dependence on a second parameter, affecting the varying sensitivity index. The simulation depicts the sensitivity index of $R_{0}$ (Equation 13) for six different locations using six different colors and sizes of circles.

**Table 2:** Description of the parameters with the values used in the deterministic modeling of Mpox transmission.

Parameter	Definition	Value	References
$N_{h}$	Total size of the human population	variables	(35)
$N_{r}$	Total size of rodent population	8 000 000	(24)
$\Theta_{h}$	Human recruitment rate	$0.3640/(52*10^{4})$ per weeks	(25)
$\Theta_{r}$	Rodent recruitment rate	0.2/52 per weeks	(48)
$\mu_{h}$	Natural death rate of human	1.5/52 per weeks	(48)
$\mu_{r}$	Natural death rate of rodent	0.002/52 per weeks	(48)
$\delta_{h}$	Weekly disease-induced death rate in the human populations	(3.6%∗7)/21	(24)
$\delta_{r}$	Weekly disease-induced death rate in the rodent populations	(0.35*7)/21	(24)
$\frac{1}{\alpha_{h}}$	Average incubation period of Mpox in the human population	13/7 weeks	(24)
$\frac{1}{\alpha_{r}}$	Average incubation period of Mpox in the rodent population	7/7 week	(48)
$\frac{1}{\gamma_{1}}$	Average number of weeks of recovery needed for infectious individuals	3 weeks	(48)
$\frac{1}{\gamma_{2}}$	Average number of weeks of recovery needed for dose-2 vaccinated individuals	1 week	assumed
$\frac{1}{d}$	Average number of weeks of recovery needed for hospitalized individuals	3 weeks	assumed, (48)
$\frac{1}{\gamma_{h}}$	Average number of weeks between symptom onsets and hospitalization	approx. 1 week	(59)
$\frac{1}{\gamma_{r}}$	Average number of weeks of recovery needed for infectious rodent	3 weeks	(48)
h	Proportion of hospitalization	3.2 to 9.4%	(60)
ϕ	Smallpox or dose-1 vaccine coverage rate	variables	(58)
σ	Dose-2 vaccine coverage rate	variables	(58)
τ	Smallpox or dose-1 vaccine failure rate	0.15 variables	(58)
$c_{0}$	Baseline contact rate among the overall population	10.8	(24)
$k_{1}$	Proportion of contacts within the human in overall contacts	0.6	(48)
$k_{2}$	Scaling factor of contact rate among the rodent compared to baseline contact	1.3	(48)

**Table 3:** Population size and fitted parameters in deterministic model fitting.

Location	$N_{h}$	$c_{0}$	$k_{1}$	$k_{2}$	$\alpha_{h}$	$\alpha_{r}$	τ
World	7,795,237,030	3.2000	0.2839	0.6499	0.9845	0.7550	0.0000
North America	592,072,212	2.7679	0.6455	0.9726	0.7843	0.6620	0.0000
South America	430,759,766	2.9452	0.8640	1.2464	0.5787	0.6235	0.0000
Europe	747,636,026	6.3535	0.8873	0.4026	1.0000	0.9988	0.0000
Brazil	212,559,417	4.3212	0.9602	0.7526	1.0000	0.7217	0.0000
USA	331,002,651	2.3433	0.7978	1.2046	1.0000	0.9989	0.0000

**Table 4:** Variable parameters and errors in deterministic model fitting.

Deterministic model fitting	Variable parameters				Test errors and test domain			
Location	η	ϕ	σ	h	MAE	RMSE	nRMSE	Test domain
World	$10^{-1}$	0.35	0.0010	0.022	760.90	1283.30	1.16	Feb. 11–June 3 (2023)
North America	$10^{-1}$	0.35	0.0012	0.032	258.77	494.07	9.50	Feb. 18–May 27 (2023)
South America	$10^{-1}$	0.35	0.0012	0.032	139.09	215.21	7.17	Feb. 18–May 27 (2023)
Europe	$10^{-1}$	0.35	0.0010	0.022	650.52	1136.3	45.45	Feb. 11–June 3 (2023)
Brazil	$10^{-1}$	0.35	0.0012	0.032	51.91	81.14	7.38	Feb. 18–May 27 (2023)
USA	$10^{-1}$	0.35	0.0012	0.032	107.02	168.91	3.31	Feb. 18–May 27 (2023)

For our Mpox model (Equations 5 and 6), we analyze the sensitivity index to its associated parameter for six locations to find the most sensitivity parameters in Figure 8.

### 3.3 Data-driven models

**Figure 9:**
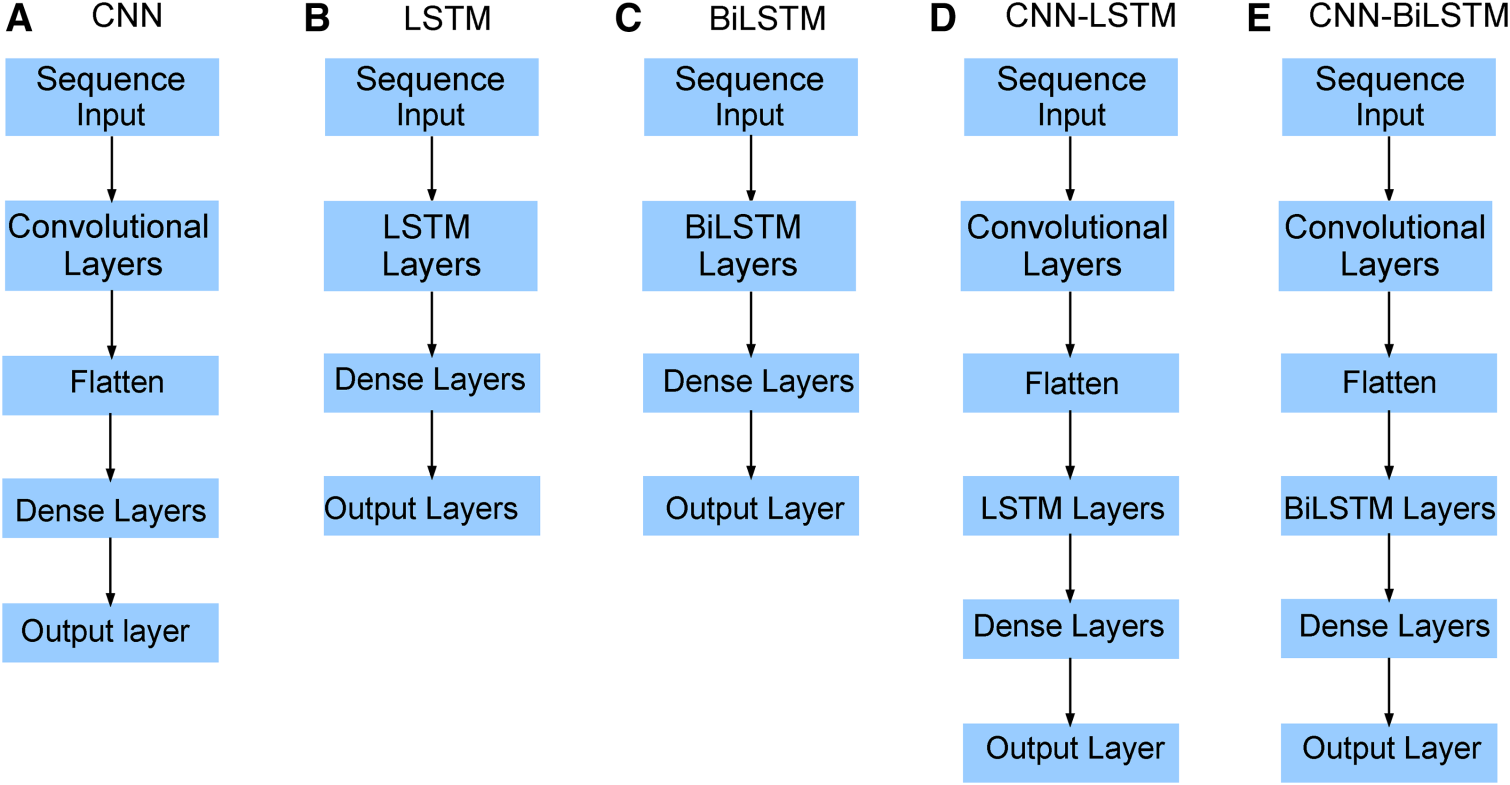
Neural network architecture: CNN, LSTM, BiLSTM, CNN-LSTM, and CNN-BiLSTM architectures involve neural network layers for feature extraction on input data. Model descriptions for time series prediction problems from sequences of data are provided in the Appendix (Tables A4–A9).

We have now introduced two statistical time series models (ARIMA and exponential smoothing) and five deep learning models (Figure 9). The primary objective of these models is to analyze Mpox data/patterns and make predictions.

#### 3.3.1 ARIMA model

ARIMA is a widely used time series model that is suitable for all kinds of data, including changing trends, seasonality, periodic changes, and random disturbances, i.e., it deals with non-stationary time series. Suppose $y_{t}$ is the actual observation and dth difference is $Y_{t}=\Delta^{d}y_{t}$. ARIMA is represented as ARIMA(p,d,q), where p is the autoregression order, d is the degree of difference, and q is the moving average order. The ARIMA model addresses non-stationary time series by modeling the difference stationary time series using an ARMA model (44). It can function as an ARMA, AR, I, or MA model. The AR(p) model linearly relates the current value or observed value $y_{t}$ at time t of the time series to its past values $y_{t-1},y_{t-2},\ldots ,y_{t-p}$ and current residuals $\epsilon_{t}$, while the MA(q) model linearly relates the current value of the time series to its current $\epsilon_{t}$ and past residual $\epsilon_{t-1},\epsilon_{t-2},\ldots ,\epsilon_{t-q}$ values. The ARIMA model is basically an ARMA model fitted on d-th order differenced time series such that the final differenced time series is stationary. $Y_{t}$ is p’th order autoregressive process, written AR(p) and is defined as

(15) $$Y_{t}=\phi_{1}Y_{t-1}+\phi_{2}Y_{t-2}+\ldots +\phi_{p}Y_{t-p}+\epsilon_{t}.$$

Furthermore (Equation 15), $Y_{t}$ constitutes a general linear process when expressed as $Y_{t}=\epsilon_{t}+\psi_{1}\epsilon_{t-1}++\psi_{2}\epsilon_{t-2}+\cdots$, with the condition that $\sum_{i=1}^{\infty }\psi_{i}^{2}<\infty$. When this equation has only a finite number of non-zero ψ coefficients, it is referred to as a moving average process. Specifically, the moving average of order q is (Equation 16):

(16) $$Y_{t}=-\theta_{1}\epsilon_{t-1}-\theta_{2}\epsilon_{t-2}-\ldots -\theta_{q}\epsilon_{t-q}+\epsilon_{t}.$$

Finally, $Y_{t}$ is an autoregressive moving average of orders p and q, written ARMA(p,q), if it can be written

(17) $$Y_{t}=\phi_{1}Y_{t-1}+\phi_{2}Y_{t-2}+\ldots +\phi_{p}Y_{t-p}+\epsilon_{t}-\theta_{1}\epsilon_{t-1}-\theta_{2}\epsilon_{t-2}+\ldots -\theta_{q}\epsilon_{t-q},$$

where the autoregressive and moving average parameters are ϕ and θ, respectively. Note that Equation 17 represents the ARIMA(p,q,d) model with $y_{t}$, where the d-th order difference $Y_{t}=\Delta^{d}y_{t}$ is substituted into Equation 17. We have used the auto_arima function to fit the ARIMA model for the univariate time series according to a provided information criterion (either AIC, BIC, or HQIC).

#### 3.3.2 Exponential smoothing model

The Exponential Smoothing model is a simple but effective method for forecasting time series data. It depends more on the most recent observations for predicting future time series values compared to older observations. The smoothing parameter α controls the weight given to the most recent observation vs. the previous forecasted value, and it is typically chosen by optimization techniques to minimize the forecast error. The basic Exponential Smoothing model, as denoted by Equation 18, is presented as follows:

(18) $${\overset{´}{y}}_{t+1|t}=\alpha y_{t}+(1-\alpha ){\overset{´}{y}}_{t|t-1},\;\text{\textbackslash{},for}\;t=1,\cdots ,T,$$

where, ${\overset{´}{y}}_{t+1|t}$ is the one-step-ahead forecast for the next time period t+1, $y_{t}$ is the actual observation at time t, ${\overset{´}{y}}_{t|t-1}$ is the forecast for time period t based on the information available up to time t−1, and α is the smoothing parameter, which takes values between 0 and 1 and controls the weight given to the most recent observation.

#### 3.3.3 CNN, LSTM, and bidirectional LSTM models

CNN is short for Convolutional Neural Network, which is a deep learning algorithm primarily used for image and video processing, as well as other types of data. For time series forecasting, a 1D-CNN architecture is used, consisting of an input layer, convolutional layer, pooling layer, flattened layer, fully connected layer, and output layer. The convolutional layers apply filters to the input data to identify features, while the pooling layers reduce the data’s dimensions to reduce computation. A flattened layer converts the reduced-size feature map into a one-dimensional array. Finally, the fully connected layers perform classification or regression based on the input data’s features. We have used ReLU as the activation function in the convolutional layer.

The Long Short-Term Memory (LSTM) model architecture is a type of recurrent neural network (RNN) designed to handle the vanishing gradient problem often encountered in traditional RNNs. LSTM networks have a complex structure that allows them to capture long-term dependencies in sequential data. Briefly, the basic structure of the LSTM unit has a memory cell, and three primary gates: an input gate, an output gate, and a forget gate. These gates determine whether to allow information to pass through the cell or forget it. The input gate decides which information from the current input and the previous hidden state should be passed to the current cell state, while the forget gate determines which information from the previous cell state should be retained or forgotten. Finally, the output gate determines the amount of output that should be passed to the next LSTM cell or the final output. The internal architecture of the LSTM model can be observed in Esmail et al. (54).

Unlike standard LSTM, the bidirectional LSTM (BiLSTM) is a recurrent neural network (RNN) architecture that processes sequential data in both forward and backward directions. It combines two LSTMs, one processing the input sequence in the forward direction and the other in the reverse direction. By considering each element’s past and future context in the sequence, BiLSTM can capture more comprehensive information. The main advantage of using BiLSTM is that they can capture both past and future context for each element in a sequence, allowing the model to make more informed predictions. However, BiLSTMs also have some limitations. They require processing the entire input sequence in both forward and backward directions, which can be computationally expensive and time-consuming. Overall, bidirectional LSTMs provide a powerful tool for capturing context and dependencies in sequential data by considering both past and future information.

#### 3.3.4 Hybrid deep learning models (CNN-LSTM, CNN-bidirectional LSTM)

The hybrid deep learning model, CNN-LSTM, combines the strengths of CNNs and LSTM networks for sequence classification, including natural language processing and time series analysis. Similarly, CNN-BiLSTM is a neural network architecture that combines CNNs with BiLSTM layers. The combination of CNN and BiLSTM layers in this architecture allows the model to effectively capture both spatial and sequential dependencies in the input data. The CNN layer extracts relevant features and reduces dimensionality, while the LSTM layer captures temporal dependencies and models sequence data. The model takes input data, passes it through the CNN layer, and feeds the output into the LSTM or BiLSTM layers to produce the final output using the dense layer. Various applications such as speech recognition, sentiment analysis, and weather forecasting have successfully utilized the CNN-LSTM model. It is a powerful tool for analyzing complex sequential data requiring feature extraction and sequence modeling (Figure 9).

## 4 Results

We implement five deep learning models: CNN, LSTM, BiLSTM, hybrid CNN-LSTM, and CNN-BiLSTM prediction model to forecast the Mpox virus transmission. A 60-10-30 split in training, validation, and test data will be applied. We evaluate the performance of the data-driven models on test data. We also compare five deep learning models with the statistical time series ARIMA and Exponential Smoothing models. Finally, we evaluate model predictions with the real Mpox data.

### 4.1 Results on deterministic model

Vaccination for all individuals globally is really challenging, but vaccination is one of the effective strategies to prevent any epidemic like COVID-19. Note that the smallpox vaccine is 85% effective against Mpox due to its genetic similarity to the smallpox virus (55, 56). Since no vaccine is 100% effective (57), and people who have been Mpox vaccinated can still get Mpox, so we assume the failure rate, τ in our proposed deterministic model given in Equation (5). However, CDC (58) reported that approximately 1,233,453 people in the USA received the Mpox vaccine as of June 20, 2023, and only 23% of the population at risk has been fully vaccinated in the USA. Moreover, there are limited vaccinations in many areas of the world; for example, the Africa Centre for Disease Control stated that the continent, with a population of over 1.2 billion people, had no access to Mpox vaccines (55). Moreover, Figure 11 illustrates the significance of vaccination for the US population.

We obtained values from the literature for most biological parameters, which in this model are either constant (Table 2) or data fitting with our model (Table 3). To simulate our model, we initialize the week one infected individual by one, i.e., $I_{h}(0)=1,I_{r}(0)=1$ by ignoring the scenario of whether there were any reported cases or not. We then fit our Mpox model with the reported data and uncover the fitted parameters in Table 3 and the variable parameters in Table 4.

#### 4.1.1 Fitting deterministic model

Suppose we are given the data $\left\{(t_{1},y_{1}),\ldots (t_{n},y_{n})\right\}$, where $y_{n}$ is the n-th observed data point, and n is the total number of data points. We want to fit ηI(t) of model estimated parameter ϑ, where η is a “reporting rate.” Then the sum of squared errors (SSE) between ηI(t) and the data can be measured by

(19) $$L\;:\;=\text{SSE}(\vartheta )=\sum_{\;j=1}^{n}\|y_{j}-\eta I(t_{j}){\|}^{2}.$$

**Figure 10:**
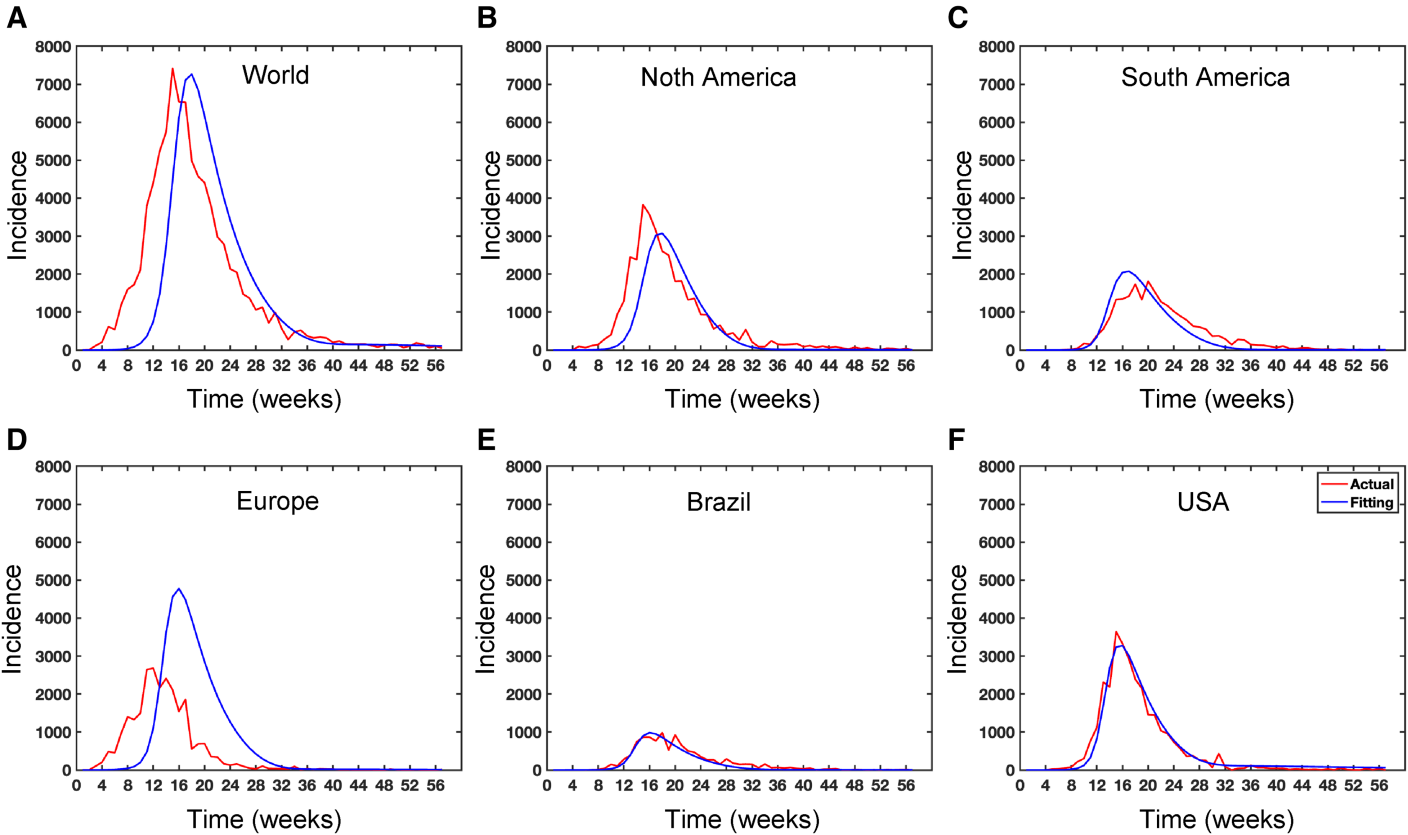
Data fitting for the Mpox deterministic model in various locations: The presented figure illustrates the Mpox data fitting of the deterministic model in various locations. As a mathematical model of the Mpox spread, we use the deterministic model shown in Figure A2. Also, Table 2 provides the initial data used for the fitting, while Table 3 contains the corresponding fitting parameters for each location. Additionally, Table 4 shows various variable parameters using the testing errors defined in Equation 2.

In the least-square fitting, we find the value $\hat{\vartheta }$ of the model parameter ϑ such that SSE(ϑ) is the minimum. We use the Matlab function *ode45* to simulate our proposed model, and Matlab’s minimization-constrained function *fmincon* takes the least-squares error function SSE(ϑ) and uses a direct search routine to find a minimum value of least squares error. In this fitting, we fix most of the parameters given in Table 2 and estimate the parameters $\vartheta =[c_{0},k_{1},k_{2},\alpha_{h},\alpha_{r},\tau ]$ using the reporting rate $\eta =10^{-1}$ on the model incidence of I. More importantly, Figure 10 presents the Mpox deterministic model fitting in various locations, and its algorithmic steps are given in Appendix A (Algorithm 1).

To investigate the particular impact of disease transmission of Mpox via inter-group contacts, we now generate colormaps based on the baseline contact rate $c_{0}$ within the overall population and the proportion of human contacts, $k_{1}$. To incorporate these numerical simulations, an outbreak is defined as in (61, 62) when the maximum value of the function I(t) within a specified time interval τ is greater than or equal to 2, represented as $\max_{t\in \tau }I(t)\geq 2$. On the other hand, the absence of an outbreak is determined when the maximum value of infected cases, I(t), within the same time frame, remains below 2, i.e., $\max_{t\in \tau }I(t)<2$, where minor fluctuations in the total count of infected individuals may arise due to numerical instability. These conditions produce the epidemic (yellow-colored) vs. non-epidemic (blue-colored) parametric regions in Figure 12.

### 4.2 Results on data-driven models

We now implement the deep learning model to the EPI weekly time series data and found that the hybrid CNN-LSTM network model performed best in both test and prediction. We choose different window sizes to group the confirmed cases in the time series data for training and testing the five neural networks: CNN, LSTM, BiLSTN, and hybrid CNN-LSTM and CNN-BiLSTM. Then, the optimal window size and hyperparameters were determined by analyzing their errors’ (MAE, RMSE, nRMSE) effect on the network’s performance. Several studies, such as the one by Sun et al. (63) in 2020, have demonstrated the practical effectiveness of the *Adam* optimizer and its favorable performance compared to other adaptive learning rate algorithms. As a result, we have implemented the *Adam* optimizer in all of our machine-learning models.

## 5 Discussion

**Figure 11:**
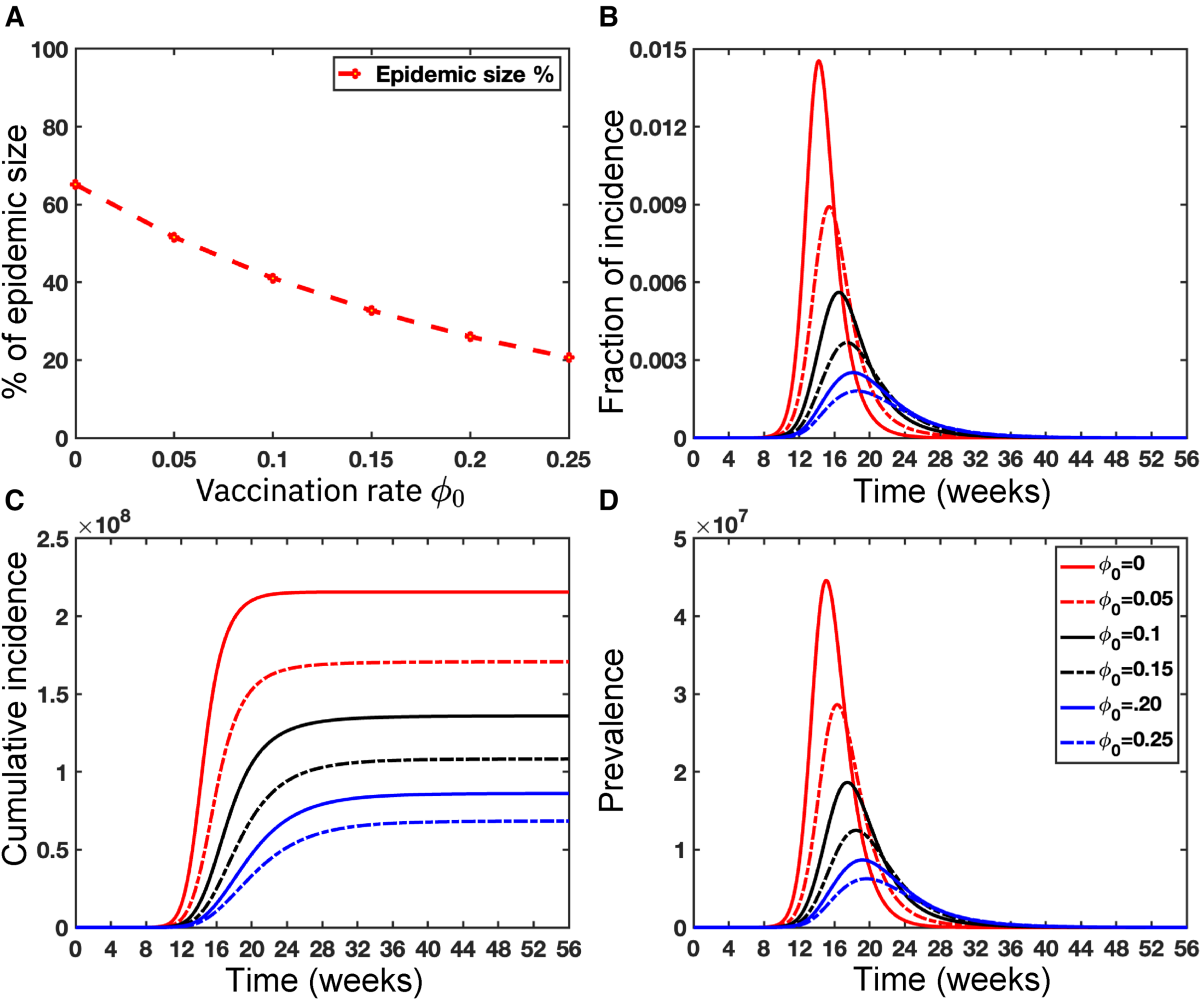
Importance of Vaccinations: This figure illustrates the significant impact of vaccination on the USA population size $N_{h}=331\text{,}002\text{,}651$. Panel (**A**) depicts the percentage of epidemic sizes for $\phi_{0}\in [0,0.25]$. Panel (**B**) demonstrates a decreasing fraction of incidence with increasing vaccination rates. Panels (**C**) and (**D**) depict the cumulative incidence and the prevalence, respectively, highlighting a substantial reduction when the vaccination rate $\phi_{0}$ increases. Overall, raising the vaccination rate ϕ of Mpox offers a solution to mitigate the risks posed by Mpox as well as other emerging infectious diseases. For these simulations, we use the parameter values given in Table 2 except the variable parameter values $\beta_{hh}=5.5,\beta_{rr}=2.5,\beta_{hr}=\beta_{rh}=2.0251$. For each simulation, we also consider the initial conditions $I_{h}(0)=I_{r}(0)=1,S_{h}(0)=N_{h}-I(0)-V_{1}(0),E_{h}(0)=H(0)=V_{1}(0)=V_{2}(0)=R_{h}(0)=E_{r}(0)=R_{r}=0,S_{r}(0)=N_{r}-I_{r}(0)$, where $N_{r}=8\text{,}000\text{,}000$. We run simulations over the time range τ=[0,65] with step size Δτ=0.1.

**Figure 12:**
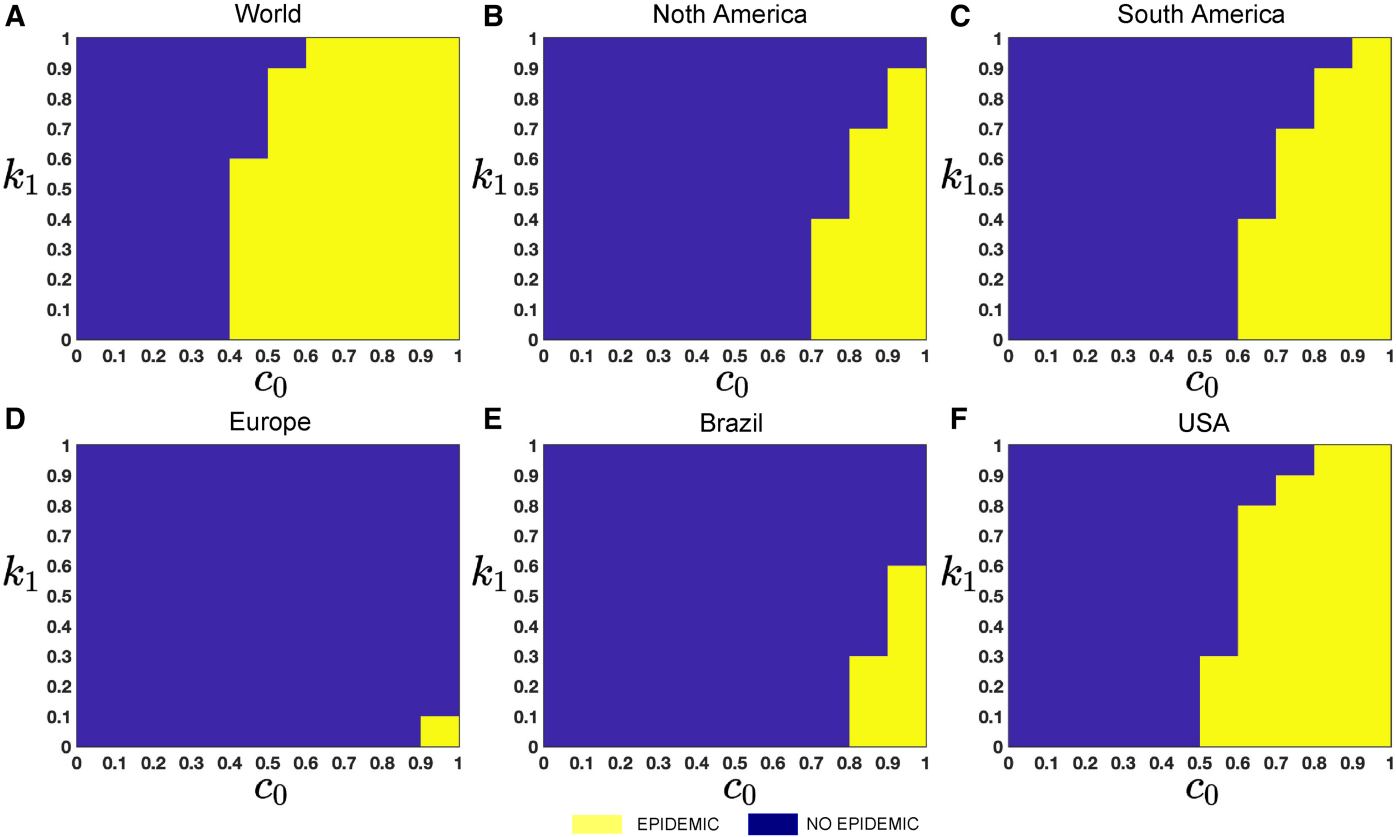
Epidemic (yellow colored) *vs.* non-epidemic (blue colored) parametric regions depending on $c_{0}$ and $k_{1}$: The figure shows the colormaps for the range of $c_{0}=[0,1]$, and $k_{1}=[0,1]$ with step size $\Delta c_{0}=\Delta k_{1}=0.1$. The epidemic region is depicted in yellow in the figure, while the non-epidemic parametric region is represented in blue. For these simulations, we use the parameter values given in Table 2, fitted parameter in Table 3, and variable parameters value in Table 4. This figure also illustrates that with these epidemiological parameters, it is possible to control the epidemic by adjusting the baseline contact rate $c_{0}$ and the proportion of contacts within the human population $k_{1}$ in overall contacts. For each simulation, we also consider the initial conditions $I_{h}(0)=I_{r}(0)=1,V_{1}(0)=N_{h}*(1/100),S_{h}(0)=N_{h}-I(0)-V_{1}(0),E_{h}(0)=H(0)=V_{2}(0)=R_{h}(0)=E_{r}(0)=R_{r}=0,S_{r}(0)=N_{r}-I_{r}(0)$, where $N_{r}=8\text{,}000\text{,}000$. We run simulations over the time domain τ=[0,65] with step size Δτ=0.01.

**Figure 13:**
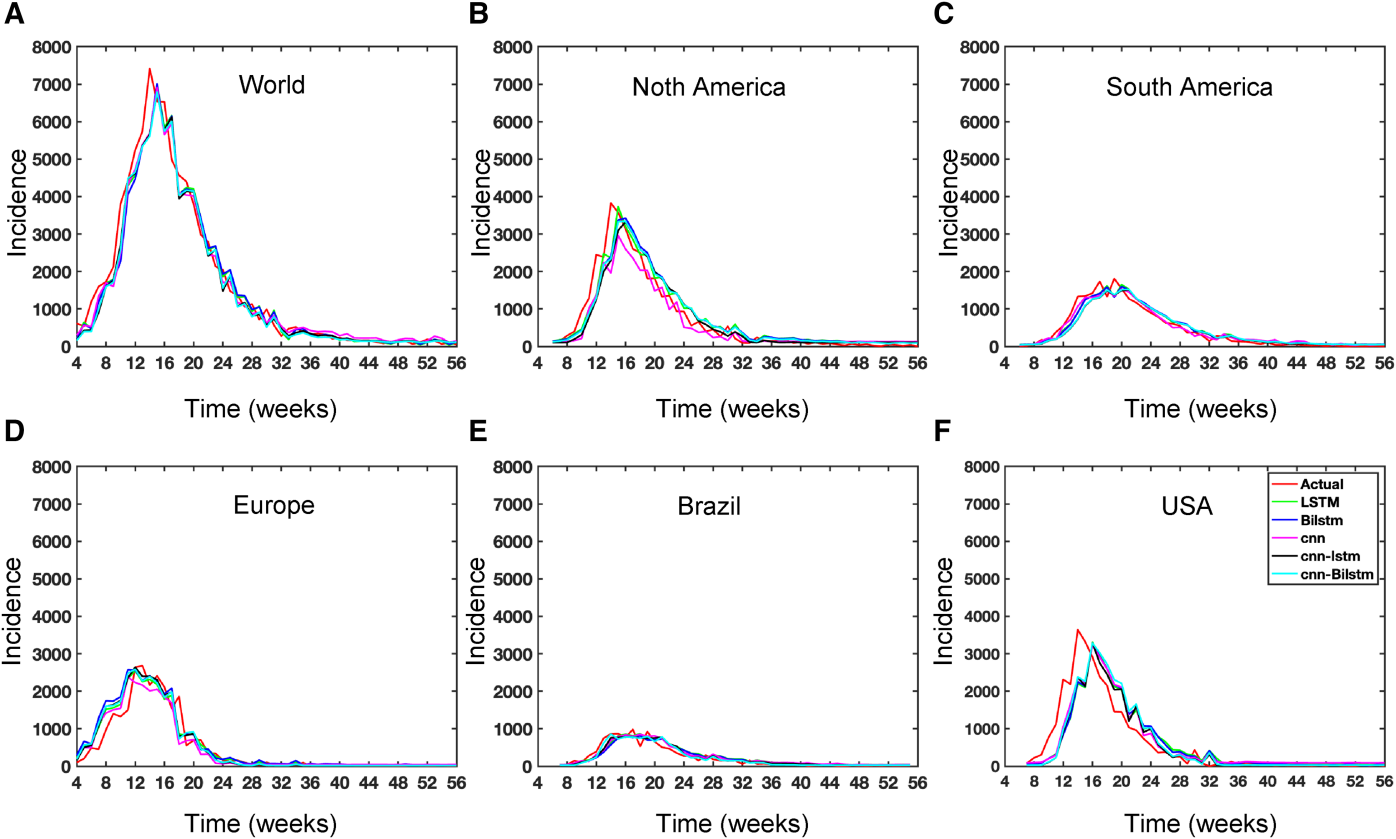
Deep learning model training for Mpox incidence in different locations: This figure shows the predicted vs. actual incidence per week in six different locations worldwide using five different deep learning models. We use 60% for the training dataset with 10% for validation during the model building (details given in the appendix) and then utilize the trained model to predict the incidence (number of new cases) per week for the entire time series data spanning from 2023-02-11 to 2023-06-03. For example, in the World and Europe time series data, the model was trained on the data from 2022-05-07 to 2023-02-04 (week 0 to week 39), and the predictions were then extended to cover the entire time whole period from 2022-05-07 to 2023-06-03 (week 0 to week 56) and compared with the actual data. The actual data are displayed in red in the panel, while the predictions from various deep learning models, including CNN, LSTM, BiLSTM, CNN-LSTM, and CNN-BiLSTM, are presented in magenta, green, blue, black, and cyan, respectively. These models were also evaluated using test data from 2023-02-11 to 2023-06-03 (week 40 to week 56), with the model predictions given in Figure 14 and subsequently used to predict the next eight weeks from 2023-06-10 to 2023-07-29 (week 57 to week 64) in Figure 15. Similarly, we optimized the model for other locations as provided in the Appendix C.

**Figure 14:**
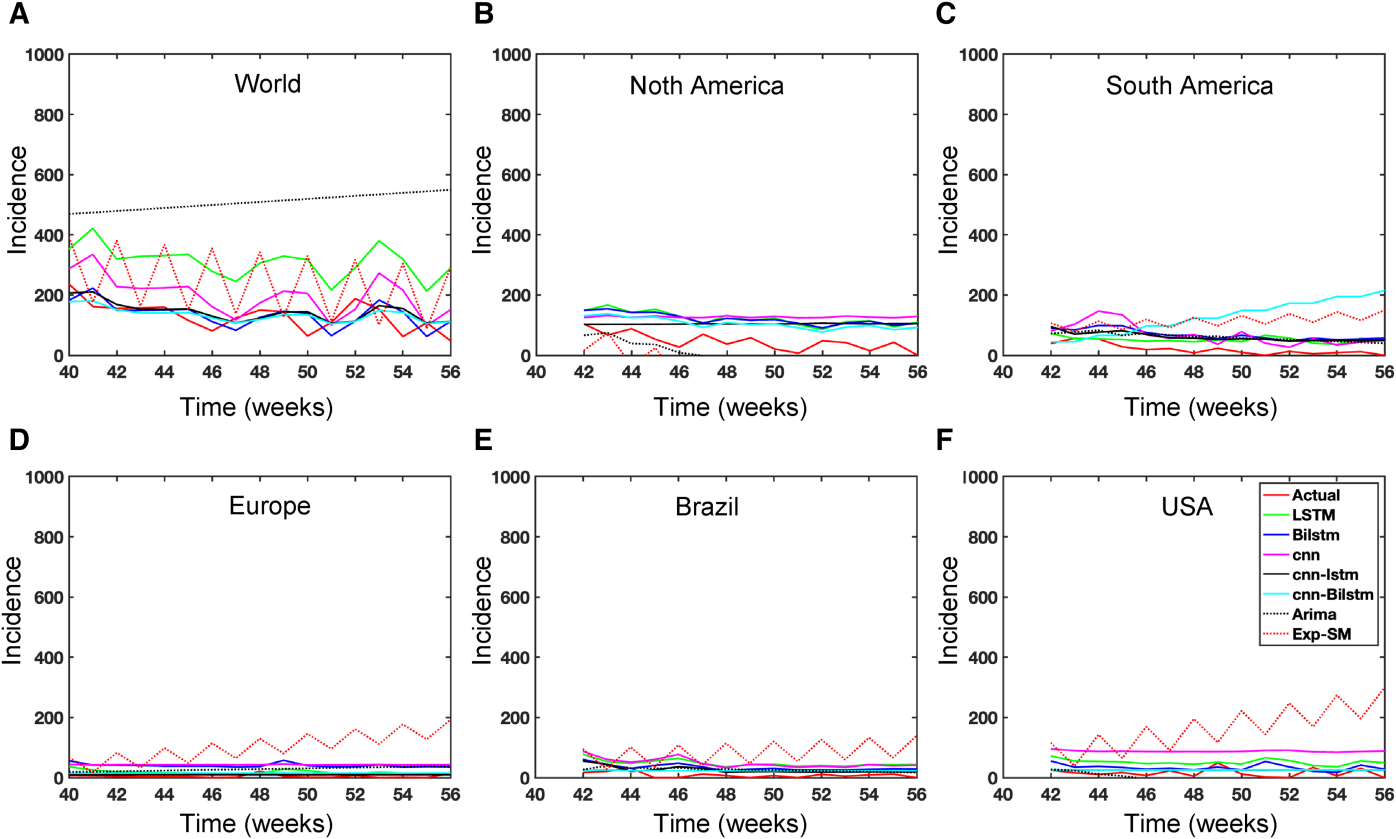
Data driven model on test data for Mpox incidence across different locations.

**Figure 15:**
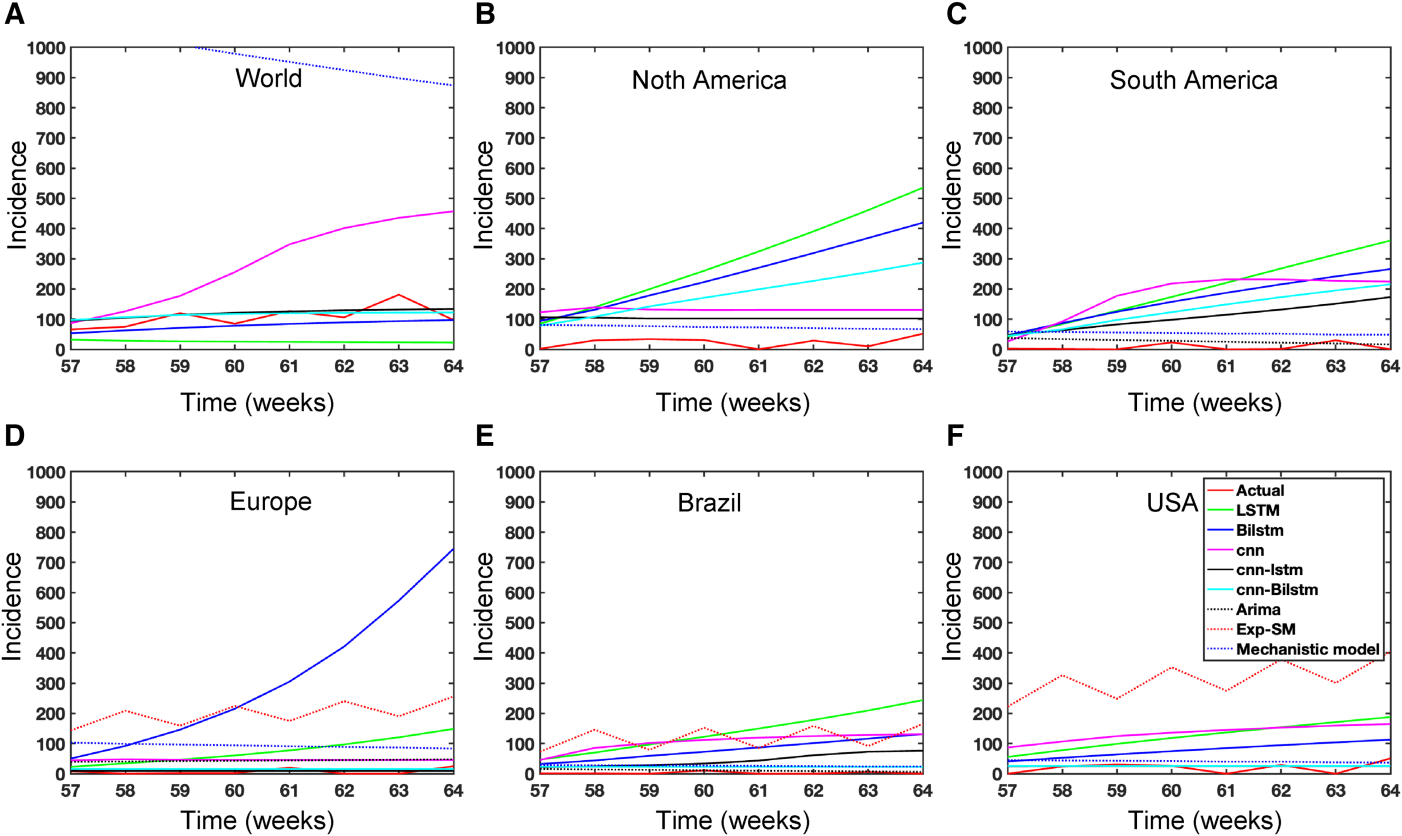
Mpox predictions of different locations using data-driven and deterministic models. Appendix C contains the data-driven model construction process, along with error measurements on the test data. Additional details, i.e., model parameters and model construction are provided in Tables A1, A2, A3, A4, A5, A6, A7, A8, and A9. Moreover, the deterministic model used the same parameter values described in Figure 10.

While recent deep learning studies on the Mpox approach stayed limited to classifying or diagnosing Mpox, for example, (64) and many more, our study modeled to explore the dynamics of disease transmission. In our modeling framework, we have done model fitting in Figure 10 and made predictions in Figure 15 and found important epidemiological parameters in Table 3. More importantly, our findings of the deterministic model (Equation 5) demonstrate that with an increasing vaccination rate, the percentage of the epidemic size decreases, as depicted in Figure 11. This result also highlights the importance of vaccination for disease control and prevention. The findings presented in Figure 12 indicate that effective control of the epidemic can be achieved by making strategic modifications to the baseline contact rate $c_{0}$ and the proportion of contacts within the human population $k_{1}$, as depicted in the accompanying Figure. Additionally, we have constructed the deep learning models and presented a comprehensive visualization of the entire time series dynamics in Figure 13. Next, Figure 14 depicts the disease dynamics observed on the test set for the data-driven models across various regions: (A) World, (B) North America, (C) South America, (D) Europe, (E) Brazil, and (F) USA. This figure also reveals that the deep learning models, namely CNN, LSTM, BiLSTM, CNN-LSTM, and CNN-BiLSTM, closely align with the reported incidence dynamics, capturing the trends effectively. However, the ARIMA and Exponential smoothing models deviate from the real incidence on test data, particularly on the World data.

In the context of the prediction shown in Figure 15, the hybrid deep learning models, CNN-LSTM and CNN-BiLSTM, exhibit good performance compared to other models across all test dynamics, demonstrating their potential for accurate disease prediction and capturing the complexities of the Mpox outbreak in different regions. Notably, most of the predictive models indicate that the Mpox disease is currently in a decline phase. The predictive trend remarkably mirrors the real data. However, it is worth noting that some models, for example, CNN, LSTM, BiLSTM, and CNN-BiLSTM indicate the possibility of another peak in the disease’s trajectory in selected geographical regions. In terms of overall performance, it is evident that both CNN-LSTM and deterministic models exhibit better predictive capabilities. Their accuracy (Table A10) and effectiveness in forecasting Mpox disease dynamics appear to stand out among the various models considered in this analysis.

### 5.1 Modeling limitation

The deterministic model is designed to incorporate explainability and account for real-world scenarios, although it does rely on specific assumptions. In contrast, data-driven modeling offers less explainability in their predictions but demonstrates good predictive capability when applied to time series data. We normalized the complete time series dataset using a data normalization approach, which involved selecting max and min values from each location’s entire time series. Note that this normalization data is only used in deep learning modeling. Another limitation of the deep learning models is that they are trained on random initial weights during the training.

## 6 Conclusion

This paper conducted a comprehensive analysis of global Mpox univariate time series data in diverse geographical locations. We proposed a deterministic model and utilized advanced deep learning techniques such as 1D-CNN, LSTM, BiLSTM, hybrid CNN-LSTM, and CNN-BiLSTM, alongside statistical time series models like ARIMA and exponential smoothing, to gain deeper insights into the Mpox disease dynamics. Moreover, the spatial pattern analysis of global Mpox data from May 1, 2022, to May 31, 2023, offered insights into the geographical distribution of the disease, helping public health authorities and policymakers focus on areas with higher risks. Our deterministic model highlighted the critical role of vaccination rates in flattening the curve of infection dynamics and influencing the basic reproduction number. It underscored the importance of increasing vaccination among susceptible populations to control disease transmission effectively. Through data fitting, we estimated crucial epidemiological parameters within our proposed deterministic model. The results showed the importance of reducing contact rates in high-risk groups to mitigate the disease outbreaks. Additionally, these findings contributed to a comprehensive understanding of disease dynamics across diverse locations and informed targeted intervention strategies for controlling and mitigating infectious disease outbreaks. Furthermore, our deterministic and data-driven models extended their utility by providing short-term (eight weeks) predictions across various geographical locations, including the World, USA, Brazil, and three continents: North America, South America, and Europe. The findings, verified by real data, suggested that Mpox is approaching its decline phase as of July 29, 2023.

## 7 Computer configuration

We performed all experiments in this study using Python programming language on Google Colab with the TensorFlow and Keras libraries through the Google Chrome browser and MATLAB. Computer info: MacBook Pro; Chip: AppleM2 Pro; Memory: 16 GB.

## Data Availability

The original contributions presented in the study are included in the article/Supplementary Material, further inquiries can be directed to the corresponding author.

## Appendix

The purpose of this appendix is to present a few results that are outlined in the main manuscript.

### B. Algorithm for data fitting via least squares estimation in the deterministic model

**Algorithm 1:** Parameter Estimation for a Deterministic Model

**Data:** Observations: The reported data we want to fit the model; Deterministic model: The model is defined in (Equation 5); Objective function: The Sum of Squared Errors (SSE) is formulated in (Equation 19); Optimization algorithm: Utilize the constrained optimization function “fmincon” available in MATLAB; **Result:** Estimated Parameters: The parameter values best fit the model and the observations **1 Initialization**; 2 Choose an initial set of parameter values manually; 3 **Define a subroutine function for the deterministic model**; 4 Write down the mathematical equations that describe the deterministic model; 5 Identify the parameters that need to be initialized; 6 Identify the parameters that need to be estimated to fit the model to the data; 7 **while** *not converged* **do** 8 **Step 1: Run the subroutines for the deterministic model to calculate the incidence;** 9 **Step 2: Define the error and optimization function**; 10 **Step 3: Find the estimated parameters**; 11 Execute the optimization algorithm until convergence; 12 The final parameter values are the estimated values that provide the best fit to the observed data; 13 Check the fitting performance, compute MAE, RMSE and nRMSE using (Equation 2); 14 If necessary, re-run with different initial estimated values to improve the fitting;

### C. Parameters and hyperparameters for data-driven modeling

For data-driven modeling, we use different libraries in Python. First, we utilize auto_arima to train the ARIMA model from the pmdarima library. Next, we fit the Exponential Smoothing model to the data using the ExponentialSmoothing function. Then, we employ the Keras deep learning library to develop the neural network models.

**Table A1:** Optimized ARIMA model selection based on location and error on test data.

Area	Best model	Cov type	Coef	Std err	z-Stat	P>|z|	MAE	RMSE	nRMSE	Neg #s
World	ARIMA(1,2,0)	AR L(1)	−0.6435	0.096	−6.723	0.000	629.66	726.98	3.85	15
		sigma2	2.992e+05	4.94e+04	6.058	0.000				
N.A.	ARIMA(1,2,0)	AR L(1)	−0.7487	0.070	−10.701	0.000	93.87	110.81	1.07	10
		sigma2	1.299e+05	2.41e+04	5.401	0.000				
S.A.	ARIMA(2,2,0)	AR L(1)	−1.0548	0.092	−11.477	0.000	37.63	39.02	0.70	0
		AR L(2)	−0.5534	0.099	−5.599	0.000				
		sigma2	2.595e+04	5,883.388	4.410	0.000				
Europe	ARIMA(0,2,2)	MA L(1)	−1.1512	0.108	−10.648	0.000	24.04	25.35	1.21	0
		MA L(2)	0.7168	0.098	7.342	0.000				
		sigma2	1.077e+05	2.13e+04	5.068	0.000				
Brazil	ARIMA(2,2,0)	AR L(1)	−1.1473	0.107	−10.715	0.000	17.37	19.39	0.61	0
		AR L(2)	−0.4733	0.111	−4.271	0.000				
		sigma2	1.885e+04	3,713.771	5.075	0.000				
USA	ARIMA(1,2,0)	AR L(1)	−0.7258	0.062	−11.622	0.000	52.69	64.76	1.32	15
		sigma2	1.391e+05	2.5e+04	5.566	0.000				

**Table A2:** Optimized Exp Smoothing model selection based on location and performance analysis.

Location	SM-level-co.	SM-trend-co.	SM-S-co.	S-Periods	int.level	int.trend	MAE	RMSE	nRMSE
World	0.9087641	0.5155482	0.0049327	2	−666.97	355.01	123.66	161.00	0.85
North America	0.8481840	0.3866436	0.1319115	2	−759.83	378.67	175.03	201.67	1.93
South America	0.6496420	0.5691656	0.0424273	2	−110.01	−1.45	93.34	98.77	1.76
Europe	0.5994405	0.5927621	0.0001492	2	−458.98	245.04	98.37	109.10	5.2
Brazil	0.6983918	0.3348396	0.0172271	2	−123.28	39.91	75.90	86.52	2.70
USA	0.7322763	0.6844459	0.0778146	2	−725.14	375.83	151.18	169.82	3.47

**Table A3:** Optimized Exp Smoothing model’s initial seasonal coefficients.

Location	Periods	Season-0	Season-1	Num. of negative-values (Neg #s)
World	2	149.10610	−60.580232	0
North America	2	160.52794	−28.526291	0
South America	2	96.613117	138.40140	0
Europe	2	31.599465	−25.975047	0
Brazil	2	94.130198	22.688328	0
USA	2	185.85629	3.7643548	0

**Table A4:** Hyperparameter tuning on World data using deep learning approach.

Model building on the training data (May 7, 2022, to February 4, 2023) with 10% validation of training data				Test: Feb.11–June 3 (2023)		
Forecasting model	Hyperparameter	Range	Best	MAE	RMSE	nRMSE
CNN	window size	(2,1) to (12,1)	(2,1)			
4 conv layers with filters: 256,128,64,32	Batch size	(2, 30)	2			
and kernel size 2,1,1,1, activation = relu	learning rate	(0.01, 0.0001)	0.0001	85.29	104.82	0.55
3 MaxPooling, and with flatten	Epochs	(1,100)	10			
3 Dense layers: 512, 256, 128, and 1 output layer						
LSTM	window size	(2,1) to (12,1)	(2,1)			
1 LSTM layer with 200 neurons	Batch size	(2, 30)	5			
activation = relu	learning rate	(0.01, 0.0001)	0.001	52.60	66.39	0.35
3 Dense layers with neurons 50, 30, and 10; 1 output layer	Epochs	(1,100)	5			
Bidirectional LSTM	window size	(2,1) to (12,1)	(2,1)			
1 BiLSTM layer with 50 units	Batch size	(2, 30)	5			
activation = relu	learning rate	(0.01, 0.0001)	0.001	49.60	62.77	0.33
3 Dense layers: 50, 30, 10, and 1 output layer	Epochs	(1,100)	40			
CNN-LSTM Hybrid	window size	(2,1) to (12,1)	(2,1)			
2 conv layers with filters: 256, 128	Batch size	(2, 30)	4			
and kernel size 1, activation = relu	learning rate	(0.01, 0.001)	0.001	46.18	58.52	0.31
1 MaxPooling, and with flatten	Epochs	(1,100)	19			
1 LSTM layer with 150 units						
3 Dense layers:50, 30, 10, and 1 output layer						
CNN-Bidirectional LSTM Hybrid	window size	(2,1) to (12,1)	(2,1)			
4 conv layers with filters: 256, 128	Batch size	(2, 30)	4			
and kernel size 1,1, activation = relu	learning rate	(0.01, 0.0001)	0.001	42.44	52.81	0.28
3 MaxPooling, and with flatten	Epochs	(1,100)	80			
1 Bidirectional LSTM layer with 200 units						
2 Dense layers: 50, 10, and 1 output layer						

**Table A5:** Hyperparameter tuning on North America data using deep learning approach.

Model building on the training data (June 4, 2022, to Feb. 11, 2023) with 10% validation of training data				Test: Feb. 18–May 27 (2023)		
Forecasting model	Hyperparameter	Range	Best	MAE	RMSE	rRMSE
CNN	window size	(2,1) to (12,1)	(2,1)			
4 conv layers with filters: 256,128,64,32	Batch size	(2, 30)	2			
and kernel size 2,1,1,1, activation = relu	learning rate	(0.01, 0.0001)	0.001	82.04	86.83	0.83
3 MaxPooling, and with flatten	Epochs	(1,100)	10			
3 Dense layers: 512, 256, 128, and 1 output layer						
LSTM	window size	(2,1) to (12,1)	(2,1)			
1 LSTM layer with 150 neurons	Batch size	(2, 30)	5			
activation = relu	learning rate	(0.01, 0.0001)	0.001	77.18	84.89	0.82
3 Dense layers with neurons 50, 30, and 10; 1 output layer	Epochs	(1,100)	13			
Bidirectional LSTM	window size	(2,1) to (12,1)	(2,1)			
1 BiLSTM layer with 40 units	Batch size	(2, 30)	5			
activation = relu	learning rate	(0.01, 0.0001)	0.001	75.25	82.41	0.79
3 Dense layers: 50, 30, 10, and 1 output layer	Epochs	(1,100)	7			
CNN-LSTM Hybrid	window size	(2,1) to (12,1)	(2,1)			
2 conv layers with filters: 256, 128	Batch size	(2, 30)	10			
and kernel size 1, activation = relu	learning rate	(0.01, 0.001)	0.001	58.95	65.36	0.63
1 MaxPooling, and with flatten	Epochs	(1,100)	19			
1 LSTM layer with 40 units						
3 Dense layers:50, 30, 10, and 1 output layer						
CNN-Bidirectional LSTM Hybrid	window size	(2,1) to (12,1)	(2,1)			
4 conv layers with filters: 256, 128	Batch size	(2, 30)	4			
and kernel size 1,1, activation = relu	learning rate	(0.01, 0.0001)	0.0001	61.00	68.62	0.66
3 MaxPooling, and with flatten	Epochs	(1,100)	5			
1 Bidirectional LSTM layer with 500 units						
2 Dense layers: 30, 10, and 1 output layer						

**Table A6:** Hyperparameter tuning on South America data using deep learning approach.

Model building on the training data (June 4, 2022, to February 11, 2023) with 10% validation of training data				Test: Feb. 18–May 27 (2023)		
Forecasting model	Hyperparameter	Range	Best	MAE	RMSE	nRMSE
CNN	window size	(2,1) to (12,1)	(2,1)			
4 conv layers with filters: 256 with MaxPooling 1,128,64,32	Batch size	(2, 30)	2			
kernel size 2,1,1,1 resp., activation = relu	learning rate	(0.01, 0.0001)	0.0001	50.31	61.87	1.1
3 MaxPooling, and with flatten	Epochs	(1,100)	10			
3 Dense layers: 512, 256, 128, and 1 output layer						
LSTM	window size	(2,1) to (12,1)	(2,1)			
1 LSTM layer with 100 neurons	Batch size	(2, 30)	5			
activation = relu	learning rate	(0.01, 0.0001)	0.001	46.62	52.41	0.94
3 Dense layers with neurons 50, 30, and 10; 1 output layer	Epochs	(1,100)	13			
Bidirectional LSTM	window size	(2,1) to (12,1)	(2,1)			
1 BiLSTM layer with 70 units	Batch size	(2, 30)	5			
activation = relu	learning rate	(0.01, 0.0001)	0.001	48.67	53.96	0.96
3 Dense layers: 50, 30, 10, and 1 output layer	Epochs	(1,100)	37			
CNN-LSTM Hybrid	window size	(2,1) to (12,1)	(2,1)			
3 conv layers with filters: 512, 256, 128	Batch size	(2, 30)	4			
and kernel size 1, activation = relu	learning rate	(0.01, 0.001)	0.0001	41.58	46.53	0.83
1 MaxPooling, and with flatten	Epochs	(1,100)	19			
1 LSTM layer with 100 units						
3 Dense layers:50, 30, 10, and 1 output layer						
CNN-Bidirectional LSTM Hybrid	window size	(2,1) to (12,1)	(2,1)			
3 conv layers with filters: 512, 256, 128	Batch size	(2, 30)	4			
kernel size 1,1 resp., activation = relu	learning rate	(0.01, 0.0001)	0.0001	45.46	51.60	0.92
1 flatten	Epochs	(1,100)	5			
1 Bidirectional LSTM layer with 250 units						
2 Dense layers: 50, 10, and 1 output layer						

**Table A7:** Hyperparameter tuning on Europe data using deep learning approach.

Model building on the training data (May 7, 2022, to February 4, 2023) with 10% validation of training data				Test: Feb. 11–June 3 (2023)		
Forecasting model	Hyperparameter	Range	Best	MAE	RMSE	nRMSE
CNN	window size	(2,1) to (12,1)	(2,1)			
2 conv layers with filters: 256,128	Batch size	(2, 30)	15			
and kernel size 2,1, activation = relu	learning rate	(0.01, 0.0001)	0.001	38.24	38.62	1.84
3 MaxPooling, and with flatten	Epochs	(1,100)	10			
2 Dense layers: 512, 256, and 1 output layer						
LSTM	window size	(2,1) to (12,1)	(3,1)			
1 LSTM layer with 85 neurons	Batch size	(2, 30)	10			
activation = relu	learning rate	(0.01, 0.0001)	0.01	14.44	16.19	0.77
3 Dense layers with neurons 50, 30, and 10; 1 output layer	Epochs	(1,100)	10			
Bidirectional LSTM	window size	(2,1) to (12,1)	(3,1)			
1 BiLSTM layer with 150 units	Batch size	(2, 30)	15			
activation = relu	learning rate	(0.01, 0.0001)	0.01	35.81	36.78	1.75
3 Dense layers: 50, 30, 10, and 1 output layer	Epochs	(1,100)	40			
CNN-LSTM Hybrid	window size	(2,1) to (12,1)	(2,1)			
2 conv layers with filters: 256, 128	Batch size	(2, 30)	15			
and kernel size 1, 1 activation = relu	learning rate	(0.01, 0.001)	0.001	6.76	7.26	0.35
1 MaxPooling, and with flatten	Epochs	(1,100)	10			
1 LSTM layer with 85 units						
3 Dense layers:150, 50, 10, and 1 output layer						
CNN-Bidirectional LSTM Hybrid	window size	(2,1) to (12,1)	(2,1)			
2 conv layers with filters: 256, 128	Batch size	(2, 30)	15			
and kernel size 1,1, activation = relu	learning rate	(0.01, 0.0001)	0.001	11.53	12.14	0.58
Flatten	Epochs	(1,100)	80			
1 Bidirectional LSTM layer with 180 units						
3 Dense layers: 100, 50, 10, and 1 output layer						

**Table A8:** Hyperparameter tuning on Brazil data using deep learning approach.

Model building on the training data (June 4, 2022, to February 11, 2023) with 10% validation of training data				Test: Feb. 18–May 27 (2023)		
Forecasting model	Hyperparameter	Range	Best	MAE	RMSE	nRMSE
CNN	window size	(2,1) to (12,1)	(2,1)			
4 conv layers with filters: 256,128,64,32	Batch size	(2, 30)	2			
and kernel size 2,1,1,1, activation = relu	learning rate	(0.01, 0.0001)	0.0001	39.97	43.82	1.37
3 MaxPooling, and with flatten	Epochs	(1,100)	10			
3 Dense layers: 512, 256, 128, and 1 output layer						
LSTM	window size	(2,1) to (12,1)	(2,1)			
1 LSTM layer with 200 neurons	Batch size	(2, 30)	5			
activation = relu	learning rate	(0.01, 0.0001)	0.001	38.86	41.28	1.29
3 Dense layers with neurons 50, 30, and 10; 1 output layer	Epochs	(1,100)	13			
Bidirectional LSTM	window size	(2,1) to (12,1)	(2,1)			
1 BiLSTM layer with 200 units	Batch size	(2, 30)	5			
activation = relu	learning rate	(0.01, 0.0001)	0.0001	25.01	28.09	0.88
3 Dense layers: 50, 30, 10, and 1 output layer	Epochs	(1,100)	37			
CNN-LSTM Hybrid	window size	(2,1) to (12,1)	(2,1)			
4 conv layers with filters: 1024, 512,256, and 128	Batch size	(2, 30)	4			
1 MaxPooling with pool size 1, kernel size 1, activation = relu	learning rate	(0.01, 0.001)	0.001	13.44	15.44	0.48
1 Flatten	Epochs	(1,100)	19			
1 LSTM layer with 100 units						
3 Dense layers:50, 30, 10, and 1 output layer						
CNN-Bidirectional LSTM Hybrid	window size	(2,1) to (12,1)	(2,1)			
6 conv: 1024, 512 with MaxPooling 1, 256, 128,64, 32	Batch size	(2, 30)	4			
kernel size 1,1,1,1,1,1 activation = relu	learning rate	(0.01, 0.0001)	0.001	15.67	17.06	0.53
Single flatten	Epochs	(1,100)	5			
1 Bidirectional LSTM layer with 250 units						
3 Dense layers: 100, 50, 10, and 1 output layer						

**Table A9:** Hyperparameter tuning on USA data using deep learning approach.

Model building on the training data (June 4, 2022, to February 11, 2023) with 10% validation of training data				Test : Feb. 18–May 27 (2023)		
Forecasting model	Hyperparameter	Range	Best	MAE	RMSE	nRMSE
CNN	window size	(2,1) to (12,1)	(2,1)			
4 conv layers with filters: 256,128,64,32	Batch size	(2, 30)	2			
and kernel size 2,1,1,1, activation = relu	learning rate	(0.01, 0.0001)	0.0001	71.42	72.65	1.48
3 MaxPooling, and with flatten	Epochs	(1,100)	10			
3 Dense layers: 512, 256, 128, and 1 output layer						
LSTM	window size	(2,1) to (12,1)	(2,1)			
1 LSTM layer with 200 neurons	Batch size	(2, 30)	5			
activation = relu	learning rate	(0.01, 0.0001)	0.001	35.26	38.41	0.78
3 Dense layers with neurons 50, 30, and 10; 1 output layer	Epochs	(1,100)	13			
Bidirectional LSTM	window size	(2,1) to (12,1)	(2,1)			
1 BiLSTM layer with 250 units	Batch size	(2, 30)	5			
activation = relu	learning rate	(0.01, 0.0001)	0.001	20.21	23.79	0.49
3 Dense layers: 50, 30, 10, and 1 output layer	Epochs	(1,100)	37			
CNN-LSTM Hybrid	window size	(2,1) to (12,1)	(2,1)			
2 conv layers with filters: 256, 128	Batch size	(2, 30)	4			
and kernel size 1, activation = relu	learning rate	(0.01, 0.001)	0.001	13.44	15.44	0.32
1 Flatten	Epochs	(1,100)	19			
1 LSTM layer with 100 units						
3 Dense layers:50, 30, 10, and 1 output layer						
CNN-Bidirectional LSTM Hybrid	window size	(2,1) to (12,1)	(2,1)			
2 conv layers with filters: 256, 128	Batch size	(2, 30)	4			
and kernel size 1,1, activation = relu	learning rate	(0.01, 0.0001)	0.001	13.81	15.79	0.32
Single flatten	Epochs	(1,100)	5			
1 Bidirectional LSTM layer with 150 units						
2 Dense layers: 50, 10, and 1 output layer						

**Table A10:** Prediction errors for different models.

Location	Error	CNN	LSTM	BiLSTM	CNN-LSTM	CNN-BiLSTM	ARIMA	exp smooth	det model
World	MAE	178	81	28	26	26	600	120	860
	RMSE	213	88.7	39.5	30.3	30.6	1613	135	864
	nRMSE	1.86	0.77	0.34	0.26	0.27	14.03	1.18	7.52
NA	MAE	107	276	227	79	160	256	423	50
	RMSE	108	310	249	81	172	260	432	53
	nRMSE	2.08	5.97	4.79	1.56	3.31	5.01	8.32	1.03
SA	MAE	171	191	158	101	126	22	141	46.5
	RMSE	185	217	173	108	137	24.5	142	48
	nRMSE	6.17	7.24	5.76	3.59	4.58	0.82	4.76	1.61
Europe	MAE	38.91	68.90	311	8.80	11.9	36.5	192	85.8
	RMSE	40.07	78.5	385	9.54	12.5	37.5	196	87
	nRMSE	1.60	3.14	15.38	0.38	0.49	1.5	7.8	3.5
Brazil	MAE	104	137	78	42.3	20.7	8.3	116	24
	RMSE	107	151	84	48	21	9.7	122	24.4
	nRMSE	9.7	13.8	7.7	4.33	1.9	0.88	11.09	2.22
USA	MAE	114	105	58	14	14	154	293	24.5
	RMSE	117	112	63	17.9	18.2	156	297	27.8
	nRMSE	2.29	2.2	1.24	0.35	0.35	3.07	5.83	0.54
